# Roles of Specific Membrane Lipid Domains in EGF Receptor Activation and Cell Adhesion Molecule Stabilization in a Developing Olfactory System

**DOI:** 10.1371/journal.pone.0007222

**Published:** 2009-09-29

**Authors:** Nicholas J. Gibson, Leslie P. Tolbert, Lynne A. Oland

**Affiliations:** Arizona Research Laboratories Division of Neurobiology, University of Arizona, Tucson, Arizona, United States of America; Institut de la Vision, France

## Abstract

**Background:**

Reciprocal interactions between glial cells and olfactory receptor neurons (ORNs) cause ORN axons entering the brain to sort, to fasciculate into bundles destined for specific glomeruli, and to form stable protoglomeruli in the developing olfactory system of an experimentally advantageous animal species, the moth *Manduca sexta*. Epidermal growth factor receptors (EGFRs) and the cell adhesion molecules (IgCAMs) neuroglian and fasciclin II are known to be important players in these processes.

**Methodology/Principal Findings:**

We report *in situ* and cell-culture studies that suggest a role for glycosphingolipid-rich membrane subdomains in neuron-glia interactions. Disruption of these subdomains by the use of methyl-β-cyclodextrin results in loss of EGFR activation, depletion of fasciclin II in ORN axons, and loss of neuroglian stabilization in the membrane. At the cellular level, disruption leads to aberrant ORN axon trajectories, small antennal lobes, abnormal arrays of olfactory glomerul, and loss of normal glial cell migration.

**Conclusions/Significance:**

We propose that glycosphingolipid-rich membrane subdomains (possible membrane rafts or platforms) are essential for IgCAM-mediated EGFR activation and for anchoring of neuroglian to the cytoskeleton, both required for normal extension and sorting of ORN axons.

## Introduction

Many types of neuron-glia interactions are known to be critical in the creation of complex neural architectures. In the primary olfactory pathway of the moth *Manduca sexta*, neuron-glia interactions have been shown to underlie glial cell proliferation and migration, the guidance and sorting of olfactory receptor axons (ORNs) as they grow from the antenna toward and into their target region of the brain, and the construction of olfactory glomeruli after the axons reach that target [1]–[4]. Evidence for neuron-glia interactions is strong in other developing olfactory systems as well [3], [5]–[8], suggesting that they have broad importance for the creation of olfactory circuitry. Since the cellular details of the olfactory neuron-glia interactions differ slightly in different species and are so well characterized in developing *M sexta*, we seek to elucidate the underlying molecular interactions in this species as the basis for a thorough understanding of the bidirectional conversation between neurons and glial cells in the establishment of a complex neuropil.

We have adduced evidence for the involvement of a number of molecules in neuron-glia communication in the developing olfactory pathway of *Manduca*, including two receptor tyrosine kinases (RTKs) - epidermal growth factor receptors (EGFRs) and fibroblast growth factor receptors (FGFRs) - and two cell adhesion molecules of the Ig superfamily (IgCAMs) - neuroglian and *Manduca* fasciclin II [4], [9]–[10; Gibson, unpublished]. Taken together with relevant results in other systems [11]–[18], our studies indicate that RTKs and IgCAMs are strong candidates to underlie axon-glia interactions involved in ORN axon outgrowth, sorting, fasciculation and subsequent glomerulus development.

As part of our study of IgCAM-EGFR interactions in the developing olfactory pathway, we investigate here the possibility that this signaling interaction is influenced by the association of these molecules with membrane rafts. Membrane rafts (formerly referred to as lipid rafts; [19]) and platforms (larger aggregates of rafts) are transient membrane subdomains enriched in sphingomyelin, glycosphingolipids (GSLs), and sterols [20]–[24]. They serve as platforms for the localization and aggregation of molecular partners, modulating their function by clustering signaling partners in close proximity or, in some cases, by keeping them separated (see [25]–[28] for reviews). They also place signaling molecules in proximity to their downstream effectors. IgCAMs and RTKs (EGFRs in particular) are known to be associated with membrane rafts in a number of systems and the function of RTKs appears to be modulated by that association [29]–[39].

In *M sexta*, ORN axons include a significant glycosphingolipid (GSL) fraction in both the developing and adult olfactory systems [40]. The tendency for GSLs to cluster in membrane rafts and the growing literature describing the ability of rafts to modulate receptor tyrosine kinase activation and IgCAM interactions [29]–[39], [41] led us to ask if the GSLs we had detected on ORN axons are components of membrane rafts which, in turn, could serve to modulate the EGFR and IgCAM interactions that have been implicated in several steps of development of the olfactory pathway [4].

As a step in dissecting the molecular bases of neuron-glia interactions critical in the formation of an olfactory pathway, we describe here studies designed to characterize the RTK and IgCAM relationships specific to axonal membrane rafts. Understanding which molecules are associated with these membrane subdomains also will serve to implicate particular signaling cascades that can be studied further. Our results indicate that pharmacological disruption of glycosphingolipid-rich membrane subdomains (possible membrane rafts or platforms) interferes with both EGFR activation and IgCAM stabilization that are essential for normal development of glomeruli.

## Materials and Methods

### Animals

*Manduca sexta* (Lepidoptera: Sphingidae) were reared from eggs on an artificial diet in a laboratory colony as described by Sanes and Hildebrand [42]. The adult antennal system develops during metamorphosis, when the larva advances through the pupal phase to become an adult moth. The pupal phase can be divided into 18 stages, each lasting 1–4 days. Pupae were staged according to features, such as eye pigmentation and leg development, visible through the cuticle under fiber-optic illumination as described by Tolbert et al. [43] and Oland and Tolbert [44].

### Preparation of cultures

#### Explants of olfactory receptor epithelium

Whole antennae were removed from the troughs of cuticle in which they develop in stage-4 female pupae; explants were prepared as described in Tucker et al. [45], except that enzymatic digestion was done at 37°C for 4 min in a Ca^2+^- and Mg^2+^-free Hanks' balanced salt solution (21250–014; Gibco, Grand Island, NY) containing 22.5 µg/ml Liberase Blendzyme 1 (Roche, Indianapolis, IN). Miniwell culture dishes were made as described previously [46]. After plating of explants, culture dishes were sealed with strips of Parafilm to prevent evaporation and incubated in a 26°C humidified incubator with room air.

### Removal of olfactory receptor neurons

In some animals, the primary olfactory center of the brain, the antennal lobe, was deprived of ORN axon input throughout development by removing the developing antenna using surgical methods described previously [44], [47]. Because ORN axons, with their cell bodies in the antennae, do not project contralaterally, the antennal lobe on the operated side received no input from ORNs [47], [48], but did receive normal input from the receptor neurons in the labial palp pit organ, which terminate in a single, readily identified glomerulus in the ventromedial part of the antennal lobe [49], [50]. The unoperated side served as the control.

### In situ depletion of membrane sterols with methyl-β-cyclodextrin (MβCD)

In females at early stage 2 or 3, insect saline alone (controls) or insect saline with MβCD (Sigma, #C-4555, 150 mg/ml) was injected into the headspace anterior to the brain. The injection sites were sealed with melted dental wax, and the animals were returned to the rearing room and allowed to develop to the desired stage.

### In vitro depletion of membrane sterols with MβCD

Explants were cultured for 6 hours, then treated with culture medium alone or culture medium plus MβCD (added at100 mM in doses to give final concentrations of 0, 0.5, 1, 1.5, and 2 mM). The cultures were incubated for an additional 24 hours, then processed for WGA and anti-horseradish peroxidase immunocytochemistry, or for anti- EGFR, neuroglian, or fasciclin II immunocytochemistry, (described below). Cells exposed to MβCD at these levels exhibited no significant signs of cytotoxicity.

### Primary antibodies for immunocytochemistry

#### Neuroglian

Mouse monoclonal antibody 3B11 against the extracellular Ig domains of *M sexta* neuroglian [51]–[54] was the generous gift of Dr. James Nardi, University of Illinois, Urbana, IL.

#### *M sexta* Fasciclin II

Mouse monoclonal antibody P1E1-1C3 (“C3,” [10], [55]), developed against the extracellular domain common to all isoforms of *M sexta* fasciclin II (MFas II), and guinea pig polyclonal antibodies specific for an extracellular region of the GPI-linked isoform of fasciclin II (GPI-FasII) and an intracellular region of the transmembrane isoform of fasciclin II (TM-FasII) [10] were the generous gifts of Dr. Philip Copenhaver, Oregon Health Sciences University, Portland, OR.

#### EGFR

We previously have shown that an antibody to a highly conserved region of the human ErbB-1 protein (#ab62, Abcam, Cambridge, MA) and an antibody to activated human EGFR (phosphorylated at tyrosine residue 845; #2231, Cell Signaling Technology, Beverly, MA) could be used to recognize the *M sexta* EGFR and the activated EGFR respectively [4], [56]. These antibodies to cytoplasmic EGFR domains were used on Vibratome sections of fixed, Triton-permeabilized brains. For immunocytochemistry of ORN cultures we used antibodies to extracellular regions of the EGFR from *Drosophila melanogaster* (Santa Cruz Biotechnology, Santa Cruz, CA; #sc-15827 and Abcam #ab49966) in order to avoid using Triton, which would have disrupted possible rafts. These antibodies bind to EGFRs regardless of activation state.

#### Horseradish peroxidase (HRP)

A rabbit polyclonal antibody to HRP (Jackson Immunoresearch, West Grove, PA, # 323-005-021) was used as a general marker for cultured neurons [57].

#### Ankyrin B

A mouse monoclonal antibody generated against a peptide corresponding to the spectrin-binding domain of human ankyrin B was purchased from Zymed Laboratories ((#33-3700, Invitrogen). In *M sexta*, this antibody recognizes a subset of ORN axons targeting a single glomerulus located dorso-posteriorly in the antennal lobe. It is used here as a marker for this axonal subset (not as a means of monitoring ankyrin B expression).

### In situ: immunocytochemistry

Pupae of various stages of development were anesthetized by cooling on ice. Brains (female, except for lectin labeling of adult male brains) were dissected under **insect saline solution** (150 mM NaCl, 4 mM KCl, 6 mM CaCl2, 10 mM HEPES, 5 mM glucose, pH 7.0, adjusted to 360 mOsm with mannitol; [58]). The perineurial sheath covering the brain was removed to aid in fixative and antibody penetration. The final step in all protocols, unless noted, was clearing the brains or sections for 15 min each first in 50% glycerol in water, then in 80% glycerol in water, and finally mounting on slides in 80% glycerol.

#### Neuroglian (3B11), *M sexta* fasciclin II (C3)

Brains were fixed on a shaker overnight (ON) at 4°C in 4% paraformaldehyde in 0.1 M phosphate buffer, pH 7.4, or in methanol/37% formalin (9∶1) at −20°C overnight. Vibratome (Technical Products International, St. Louis, MO) sectioning and immunocytochemistry were performed as previously described [4].

#### EGFR and activated EGFR (pEGFR)

The fixation protocol of Sinakevitch et al. [59] was used. Briefly, brains were dissected into 2.5% paraformaldehyde, 1% glutaraldehyde, 1% sodium metabisulfite in 0.1 M cacodylate buffer, pH 7.2, microwaved with a Pella research-grade oven (# 3450, with variable power controller #3430 and cold spot) with chilling plate at 18°C and power setting #2 for 2 min on/2 min off/2 min on/2 min off, and then fixed overnight on a shaker at 4°C. Following sectioning, brains were washed for 30 min in freshly prepared 0.01 M NaBH_4_, 0.5% sodium metabisulfite, in 0.05 M Tris-HCl, pH 7.5. Immunocytochemistry was performed as previously described [4].

#### Ankyrin B

Reproducible labeling of a unique set of ORN axons was achieved by using a high-pH fixation protocol: dissected brains were microwave-fixed in 50 mM carbonate buffer, pH 9.4, containing 2.5% paraformaldehyde, 1% glutaraldehyde, and 1% sodium metabisulfite (final pH = 10.8), followed by incubation in fixative solution ON at 4°C. Brains were embedded, sectioned, and washed for 30 min in freshly prepared 0.01 M NaBH_4_, 0.5% sodium metabisulfite, in 0.05 M Tris-HCl, pH 7.5. Sections were incubated in **Triton-containing blocking solution** [Tris-buffered saline (TBS; 20 mM Tris-HCl, 150 mM NaCl, pH 7.4) with 0.1% Triton X-100, 0.1% sodium azide, and 1% Ig-free bovine serum albumin (# 001-000-161, Jackson ImmunoResearch, West Grove, PA)] for 1 hr at room temperature (RT), then anti-ankyrin B was added at 2 µl/ml. Sections were incubated at 4°C ON, then washed 4X30 min in TBS with 0.1% Triton (TBST) and incubated with Cy3-conjugated goat anti-mouse IgG+IgM antibody (Jackson # 115-165-068) at 1∶300 in blocking solution for 2 hrs at room temperature. To amplify the signal, sections were washed and incubated with Alexa 564-conjugated donkey anti-goat (Molecular Probes) at 1∶250 in blocking solution ON at 4°C, then washed and further labeled with Syto 13 as described below. Controls for nonspecific immunolabeling were no-primary controls.

### In vitro: immunocytochemistry of MbCD-treated ORN explants

In the following protocols, in order to get as accurate a picture of molecule distribution as possible, it was necessary to use antibodies recognizing an extracellular domain of the target molecule, so that Triton permeabilization of the cell membranes could be avoided.

#### Anti-EGFR antibody

Control and MβCD-treated explants cultured for 30 hrs were rinsed in insect saline 3X5 min, fixed in 4% paraformaldehyde in PBS overnight at 4°C, washed 4X5 min in TBSA, and then incubated in blocking solution (TBSA with 0.5% fish gelatin, Sigma #G7765) for 1 hr. A mouse monoclonal antibody to an extracellular region of the *D. melanogaster* EGFR (Abcam #ab49966) was added to blocking solution at 1∶200. Dishes were incubated in primary antibody solution (80 µl/dish) overnight at 4°C in a humidified chamber, rinsed 4X5 min in TBSA, incubated in blocking solution (80 µl/dish) containing Cy3-conjugated goat anti-mouse IgG+IgM antibody (Jackson #115-165-068) at 1∶300 for 4 hr at RT, washed 4X5 min in TBSA, and mounted in TBSA.

#### Anti-neuroglian antibody

Control and MβCD-treated explants cultured for 30 hrs were rinsed in insect saline 3X5 min, fixed in 2% paraformaldehyde in insect saline overnight at 4°C, washed 4X5 min in insect saline, and then incubated in blocking solution (insect saline with 5% normal donkey serum and 1% Ig-free bovine serum albumin) for 1 hr at RT. Mouse anti-neuroglian (3B11) was diluted 1∶1,000 in blocking solution. Dishes were incubated in primary antibody solution (80 µl/dish) overnight at 4°C in a humidified chamber, rinsed 4X5 min in SIS, incubated in blocking solution (80 µl/dish) containing Alexa 488-conjugated goat anti-mouse F(ab′)_2_ antibody fragments (Molecular Probes, #A11017) at 1∶300 for 4 hr at RT, washed 4X5 min in insect saline, and mounted in insect saline.

#### Anti-fasciclin II antibody

Control and MβCD-treated explants cultured for 30 hrs were rinsed in insect saline 3X5 min, fixed in 4% paraformaldehyde in 0.1 M phosphate buffer, pH 7.4, overnight at 4°C, washed 4X5 min in TBS, and then incubated in blocking solution (TBS with 0.5% fish gelatin) for 1 hr. A mouse monoclonal antibody to MFas II (C3) was added to blocking solution at 1∶5,000. Dishes were incubated in primary antibody solution (80 µl/dish) overnight at 4°C in a humidified chamber, rinsed 4X5 min in TBS, incubated in blocking solution (80 µl/dish) containing Alexa 488-conjugated goat anti-mouse F(ab′)_2_ antibody fragments at 1∶400 for 4 hr at RT, washed 4X5 min in TBS, and mounted in TBS.

### In situ: lectin labeling of whole brains

Sectioned male brains that had been fixed on a shaker ON at 4°C in 4% paraformaldehyde plus 0.15% glutaraldehyde in 0.1 M phosphate buffer, pH 7.4, were incubated ON at 4°C in 0.5 ml lectin buffer (300 mM NaCl, 100 µM CaCl2 in 10 mM HEPES, pH 7.5) containing 2 µl (10 µg) each of Rhodamine-labeled wheat germ agglutinin (WGA, *Triticum vulgaris*) and fluorescein-labeled *Artocarpus integrifolia* lectin (Jacalin) (Vector Laboratories, Burlingame, CA) or Alexa-633-labeled WGA (#W21404) (Invitrogen, Carlsbad, CA).

### In vitro: lectin labeling of explants

Cultured explants of the olfactory epithelium of the antenna were fixed in 4% paraformaldehyde with 0.1% glutaraldehyde in 0.1 M phosphate buffer, pH 7.4 for 30 min, washed in phosphate-buffered saline (PBS), and incubated with 0.5 µg WGA-rhodamine or WGA-Alexa 633 in 1 ml PBS for 2 hr at RT. The explants were then washed 2X10 min in PBS, and mounted in PBS.

### In vitro: combined immunocytochemistry and lectin labeling

#### Lectin with anti-EGFR antibody

Explants cultured for 24 hours were rinsed in insect saline 2X5 min, fixed in 2% paraformaldehyde in insect saline for 30 min at RT, washed 2X5 min in insect saline, and then incubated in blocking solution (insect saline with 1% IgG-free BSA and 5% normal donkey serum) for 1 hr. Dishes then were incubated in a mixture of goat anti-*Drosophila* EGFR (Santa Cruz Biotechnology, #sc-15827) at 1∶1000 and WGA-rhodamine (1∶10,000) in blocking solution (100 µl/dish) for 3 hrs at RT. Controls were incubated in blocking solution minus antibodies or WGA. Dishes were rinsed 3X5 min in TBS, incubated in blocking solution (100 µl/dish) containing Cy5-conjugated donkey anti-goat antibodies (Jackson # 705-175-147) at 1∶400 for 2 hrs at RT, washed 2X5 min in TBS, and mounted in TBS.

#### Lectin with anti-*M sexta* neuroglian or anti-*M sexta* fasciclin II antibody

Explants cultured for 24 hours were rinsed in insect saline 2X5 min, fixed in 4% paraformaldehyde and 0.5% glutaraldehyde in TBS for 30 min at RT, washed 2X5 min in TBS, and then incubated in blocking solution (TBS with 1% IgG-free BSA) for 1 hr. Mouse anti-neuroglian (3B11) (1∶1000) or mouse anti-MFas II (C3) (1∶5000) were added with WGA-rhodamine (1∶10,000) to blocking solution (100 µl/dish). Controls were incubated in blocking solution minus antibodies or WGA. Dishes were incubated in primary antibody solution overnight at 4°C, rinsed 3X5 min in TBS, incubated in blocking solution (100 µl/dish) containing Alexa 488-conjugated F(ab')_2_ fragment of goat anti-mouse antibodies at 1∶400 for 2 hrs at RT, washed 2X5 min in TBS, and mounted in TBS.

#### Lectin with anti-HRP

MβCD-treated explants cultured for 30 hrs were rinsed in insect saline 2X5 min, fixed in 4% paraformaldehyde and 0.1% glutaraldehyde in PBS for 30 min at RT, washed 2X5 min in PBS, and then incubated in blocking solution (PBS with 1% IgG-free BSA) for 1 hr. Rabbit anti-HRP (1∶1,000) was added with WGA-rhodamine (1∶10,000) to blocking solution (100 µl/dish). Dishes were incubated in primary antibody solution overnight at 4°C, rinsed 3X5 min in PBS, incubated in blocking solution (100 µl/dish) containing Alexa 488-conjugated goat anti-rabbit antibody (Molecular Probes) at 1∶400 for 1 hr at RT, washed 2X5 min in PBS, and mounted in PBS.

### Labeling of cell nuclei

To render glial-cell nuclei visible, all cell nuclei were labeled with a DNA-specific tag (Syto 13; Molecular Probes, #S-7575) as described previously [4]. Glial nuclei were identified by their small size compared to neuronal nuclei and by their location either in the axonal sorting zone region of the antennal nerve where they are the only cell type present [9] or in the envelope surrounding each glomerulus [44], [60].

### In vitro: Vybrant DiI

Explants of olfactory epithelium growing in culture were labeled using a protocol adapted from Hering et al. [61]. Cultures were fixed and washed as for WGA-rhodamine labeling. Vybrant DiI (#V-22885, 1 mM in ethanol, Molecular Probes, Eugene OR), at 0.5 µl in one ml PBS was added to each dish and allowed to incorporate into cell membranes for 20 min at RT. Cultures were washed 2X10 min in PBS, incubated in PBS at 4°C for 24–48 hr to allow dye diffusion within membranes, then imaged using confocal microscopy with an upright microscope using the hanging-drop technique. After imaging, the culture dishes were placed on ice and 0.5% Triton in PBS (4°C) was added for 10 min to extract the dye from non-raft membranes. Cells were washed 4X10 min in 4°C PBS on ice to remove the Triton and then re-imaged.

### Confocal microscopy and image processing

#### Vibratome sections

Sections were viewed on a Nikon PCM 2000 laser scanning confocal system (Nikon E800 microscope equipped with argon, green He Ne, and red He Ne lasers) and Simple 32 software (Compix Inc., Cranberry Township, PA) or on a Zeiss 510 Meta equipped with argon and green and red He Ne lasers and LSM software. Optical sections were acquired at 1- to 10-µm intervals through the depth of the antennal lobe (AL) and saved as three-dimensional stacks. Confocal image stacks were projected and merged in false color using Confocal Assistant (copyrighted by Todd Brelje, distributed by Bio-Rad, Richmond, CA) or the Zeiss LSM image browser, and then imported into Corel Photopaint, where image hue, intensity, and contrast were adjusted for maximum clarity. The images were then combined into figures in Corel Draw, where annotations were added.

#### Tissue culture

Dishes were imaged as above, with optical sections acquired at 0.1 to 0.3 µm intervals. In cases where visualizing all axons or processes was needed, brightfield images also were collected.

### Counting of MFas II-positive glomeruli in control and MβCD-treated antennal lobes

Previous studies revealed that untreated antennal lobes contain 14–21 glomeruli that label with the MFas II (C3) antibody [10]. In the current study, the entirety of each AL was sectioned at 100-µm intervals with a Vibratome and imaged. Each labeled glomerulus was followed through the section and between sections, if necessary, resulting in a count of MFas II-labeled glomeruli.

### Inhibition of EGFR activity

The effects of the highly selective, cell-permeable EGFR inhibitor PD168393 [62] (#513033, Calbiochem, La Jolla, CA; IC_50_  = 700 pM) on AL development have been described previously [4]. We injected PD168393 (250 or 500 µmol in 5 or 10 µl DMSO) or DMSO alone into the headspace at early stage 5 of adult development. The animals were then returned to the incubator and allowed to develop until they reached stage 7.

### Sucrose step-gradient flotation of detergent resistant membranes

Brains from 24 animals at stages 6 and 7 (12 each male and female) were stripped of their perineureum. Antennal lobes and attached nerves were removed from the dissected brains so that they could be processed and evaluated separately from the rest of the brain. Separation of Triton soluble and Triton-resistant fractions was performed basically as previously described [63], with several modifications: 1) tissue was homogenized directly after dissection to avoid possible artifacts resulting from freezing; 2) homogenization buffer included phosphatase inhibitors (Sigma P5726) in addition to protease inhibitors (Sigma P2714); 3) additional gradient steps (25 and 35%) flanking the 30% step were added to better distinguish the Triton-resistant fractions because previous work [63; Gibson, unpublished] revealed that the 30% sucrose fraction contained the components common to membrane rafts, while the 40 and 60% fractions contained the Triton-soluble membranes and associated proteins; 4) buoyant fractions were demarcated and collected based on presence or absence of material made visible by light scattering, rather than by fixed volume. The 40 and 60% sucrose fractions were pooled.

### Immunoblotting of gradient fractions

Proteins from the sucrose gradient fractions were precipitated with −20°C acetone, resolubilized in SDS-PAGE loading buffer containing protease and phosphatase inhibitors, separated via SDS-PAGE, and transferred to PVDF membranes as described previously [4], [10], [63]. One fourth of the total protein collected (corresponding to 12 ALs with attached nerves) was loaded in a given lane. Immunoblots using antibodies to pEGFR, *M sexta* neuroglian, and the transmembrane and GPI-linked isoforms of *M sexta* fasciclin II were performed as described [4], [10], [63].

## Results

The following brief description of the olfactory pathway will provide orientation to the adult and developing pathways in *M sexta*. In the adult moth [3], [64], [65], ORN axons extend from cell bodies in the sensory epithelium of the antennae through the antennal nerve to their target glomeruli in the AL, entering the glomeruli from the nerve layer that lies external to the glomerular layer. Within the glomeruli, the ORN axons synapse with the dendrites of AL neurons whose cell bodies lie entirely outside of the neuropil, mainly in two major clusters. Each glomerulus is almost completely surrounded by an envelope of glial cells and their processes, and the glomeruli are arrayed in a single layer around a coarse central neuropil composed of AL neuron dendrites. Output neurons of the AL send their axons via discrete tracts to higher order centers in the brain.

**Figure 1:**
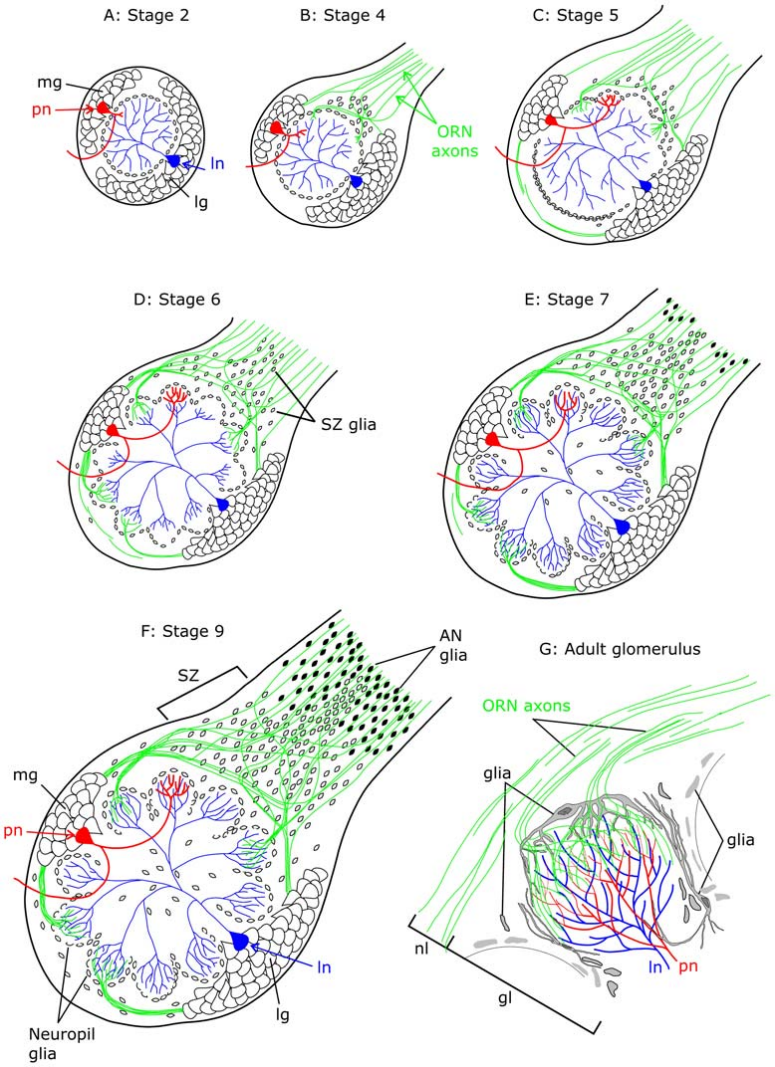
Schematic diagram of antennal lobe development in *Manduca sexta.* A: At stage 2 of development, prior to arrival of ORN axons from the antennae, the nascent AL consists of a medial group (mg) of projection neurons (pn, one shown in red), a lateral group (lg) comprising local interneurons (ln, one shown in blue), uniglomerular projection neurons, and multiglomerular projection neurons, and AL glia (small cells) surrounding a coarse neuropil. B: The first ORN axons (green) arrive at stage 4. The axons induce a subset of glial cells to proliferate and migrate outward toward the ingrowing axons to form a sorting zone at the base of the antennal nerve. C: By stage 5, ORN axons arriving at the sorting zone are induced to disassociate from other axons, change course dramatically, and refasciculate with other axons targeting common glomeruli. ORN axons penetrate the layer of glial cells, their terminal branches form glomerular arborizations called “protoglomeruli,” and the glial cells begin to migrate to surround them. Dendrites of the medial cluster projection neurons begin to extend into the forming glomeruli. D, E: During stages 6 and 7, ORN axons continue to arrive, projection neurons and now local interneurons extend their dendrites into the glomeruli, and glial cells continue to migrate to surround the glomeruli. F: By stage 9 the antennal lobe architecture is established. G: A single adult glomerulus. ORN axons traveling in the nerve layer (nl) turn sharply to innervate the apical half of a glomerulus in the glomerular layer (gl). ln and pn dendrites cross the basal border of the glomerulus, arborize, and form synaptic contacts with ORNs and each other mainly in the basal two-thirds of the glomerular neuropil. Glial cells of the simple type (75–100/glomerulus) form a sheath around the glomerulus (the processes of several are shown), while complex glial cells (<10/glomerulus) extend processes into the glomerular neuropil, arborizing in the most apical and in the basal portion of the glomerulus [137].

Development of the primary olfactory pathway of the moth occurs during the metamorphosis from larva to adult. Metamorphosis can be divided into 18 stages, each roughly a day in length [44]. ORN axons navigate toward the AL and induce the proliferation of glial cells in the entry region of the nerve at stage 4 (Fig. 1B). These glia then migrate into the base of the nerve, where they populate an axon sorting zone (SZ) during stages 5 through 7 (Fig. 1C–E). Later axons growing in from the antenna dramatically change their trajectories when they enter this glia-rich SZ, sorting into fascicles destined for individual glomeruli or subsets of glomeruli in particular regions of the AL [9]. Once in their target region, the axon terminal branches form round “protoglomeruli” during stages 5 and 6, and the array of protoglomeruli serves as the template for the mature glomerular array that is constructed during stages 6 through 9 (Fig. 1D–F) [66], [67]. Neuropil glial cells migrate between protoglomeruli and extend processes to stabilize the protoglomeruli, as the dendrites of AL neurons grow in and begin participating in synapses at stage 7 [68].

### Identification of GSL-rich membrane subdomains and associated molecules on ORN axons

As a first step in determining whether interaction among the signaling molecules implicated in developmental events in the olfactory pathway of *M sexta* could be modulated by inclusion in or exclusion from rafts, we sought to determine the pattern of membrane rafts on ORN axons.

Previous experiments [40], [63] established the presence of detergent-resistant membranes containing GSLs, sphingomyelins, and a GPI-linked protein, all characteristic components of membrane rafts, in developing (stage-7) brains of *M sexta*. We also have shown in *M sexta* that WGA labels one or more GSLs on ORN axons in fixed brain sections [40].Because GSLs are known to be concentrated in membrane rafts, we used WGA as a probe for rafts.

**Figure 2:**
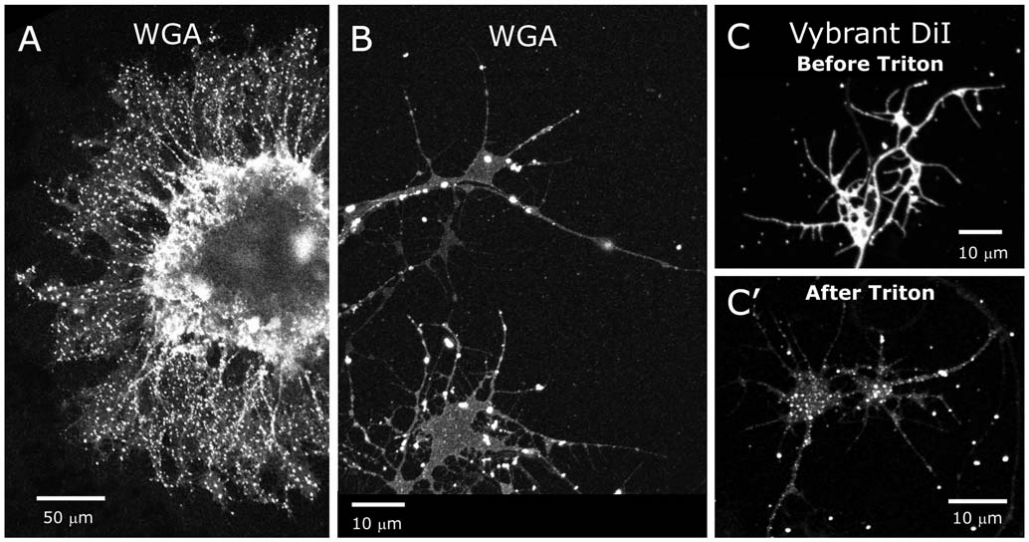
Glycosphingolipids and detergent-resistant patches on ORN axons. A: Explants of antennal olfactory epithelium labeled for glycosphingolipids (GSLs) using the lectin wheat germ agglutinin (WGA). Patchy labeling suggests that GSLs are confined to membrane subdomains. B: Higher magnification of axons and growth cones of another 24-hour culture. Patches of WGA labeling extends into growth cone filopodia. C: Individual neurons labeled with Vybrant DiI, which uniformly labels cell membranes. C′: Neurons re-imaged after treatment with 0.5% Triton X-100 at 4°C to extract the dye from phospholipid membranes but not from detergent-resistant membranes show patchy labeling indicative of membrane rafts.

To allow resolution at the level of single axons, we used WGA to label axons extending from explants of the antennal sensory epithelium from stage-4 animals that had been cultured for 24–48 hr. WGA labeling appeared in the cell bodies and along the length of the ORN axons and into their growth cones, including the filopodia. The axons displayed patchy labeling, consistent with localization of these GSLs to subdomains of axonal plasma membranes (Fig. 2A, B). The sizes of the patches varied from approximately 0.3 to 1.4 µm. Rafts are now defined to have diameters of just 10–200 nm (Keystone Symposium on Lipid Rafts and Cell Function; [19]), but published reports of confocal imaging studies using fluorescent raft components [69] report sizes (∼1 µm) similar to those found here and likely reflect amplification of apparent size by attachment of multiple fluorescent molecules and/or aggregation of smaller raft domains.

**Figure 3:**
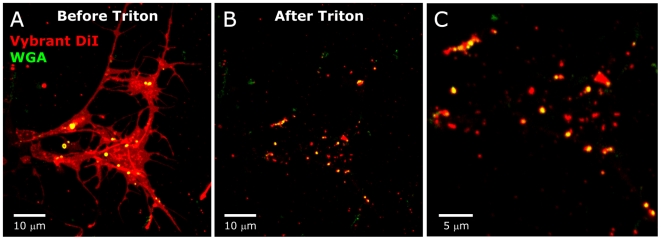
WGA colocalizes with Triton-resistant Vybrant DiI. A: Explants of antennal sensory epithelium. Vybrant DiI (red) and WGA-Alexa 633 (green). B: Re-imaging after treatment with 0.5% Triton at 4°C shows WGA labeling only where Triton-resistant Vybrant DiI remains. C: Higher magnification reveals a population of Triton-resistant Vybrant DiI-labeled patches with no detectable WGA labeling.

To test the hypothesis that the WGA-labeled (and therefore GSL-rich) patches were membrane rafts or raft aggregates, we used an additional technique, described by Hering et al. [61], which takes advantage of the operational definition of rafts as membrane subdomains that are resistant to extraction by Triton X-100 at 4°C [21]–[23]. Cultured neurons were fixed and labeled with Vybrant DiI, a lipophilic dye, followed by extraction of the dye from most phospholipid moieties (but not from detergent-resistant membrane subdomains) with Triton X-100 at 4°C. The resulting patchy labeling (Fig. 2C, C′) is similar to that seen for WGA labeling. In preparations doubly labeled with Vybrant-DiI and WGA, the WGA was always co-localized with Vybrant DiI patches (Fig. 3A–C), suggesting that the GSLs previously shown to be ligands for WGA are indeed components of membrane subdomains compositionally similar to rafts. *Not all of the Vybrant-DiI patches were associated with WGA*, however (Fig. 3C), possibly indicating that the ORN axons have multiple membrane subdomains of different lipid composition that house different molecular species, as has been found in several vertebrate cell types [70]–[75]. Because of the difficulty inherent in proving the existence and characteristics of membrane rafts in cell membranes [69], we will simply refer to the detergent resistant, GSL-rich patches seen in our ORN explant cultures as glycosphingolipid-rich membrane subdomains (gMSDs).

**Figure 4:**
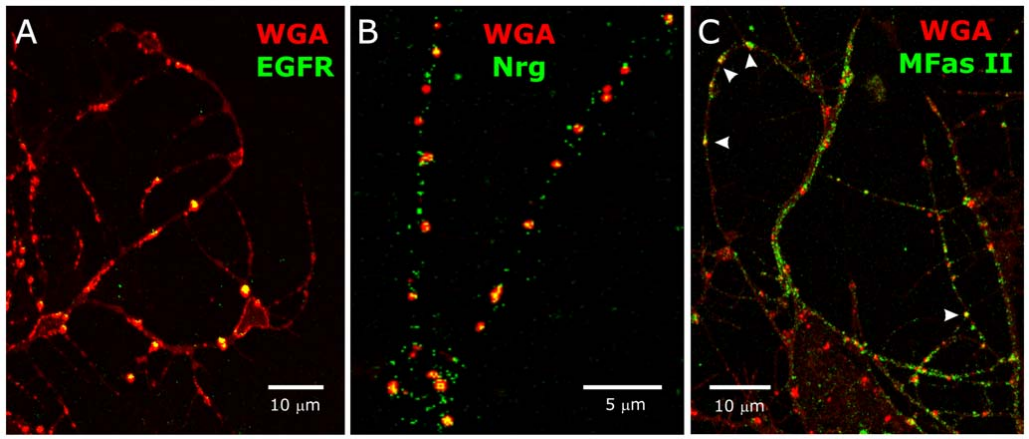
Co-localization of WGA-labeled patches and various signaling molecules expressed by ORN axons. 24 hrs *in vitro*. ORN axons extend from explants outside of the field of view. A: Co-labeling with WGA-rhodamine (red) and an anti-EGFR antibody (sc-15827, green): EGFRs are localized exclusively to WGA-labeled domains. B: Co-labeling with WGA-rhodamine (red) and an anti-neuroglian (Nrg) antibody (3B11, green): neuroglian molecules exist both in and out of WGA-labeled patches. C: Co-labeling with WGA-rhodamine (red) and an anti-MFas II antibody (C3, green): most MFas II molecules are located outside of WGA-labeled patches. Arrowheads show regions of co-localization.

We then asked whether IgCAMs and/or EGFRs co-localized with the gMSDs described above. Aldehyde-fixed (see Methods section) 24–48 hr ORN explant cultures were labeled with WGA-rhodamine, as a gMSD marker, and antibodies to the extracellular domains of EGFR, neuroglian, or *M sexta* fasciclin II (MFas II). EGFRs appeared to co-localize with WGA-labeled patches (Fig. 4A). Small neuroglian-positive puncta were detected inside most WGA-labeled patches but also in areas not labeled with WGA (Fig. 4B). MFas II (C3) immunoreactivity was found over most of the lengths of the axons, but infrequently in the WGA-labeled patches (Fig. 4C). Figure 4 indicates that EGFRs may be normally confined to WGA-labeled gMSDs, whereas neuroglian and MFas II may be found in or out of those domains. In light of our results with WGA-Vybrant DiI co-labeling, it is possible that the IgCAM molecules not co-localized with WGA were associated with other membrane subdomains which do not bind WGA, but we were unable to test this *in vitro* because we were not able technically to label for IgCAMs and Vybrant DiI-positive detergent-resistant patches in the same preparations, and we have found no lectin label for the gMSDs not recognized by WGA [40].

### Sucrose-gradient flotation of membrane subdomains

**Figure 5:**
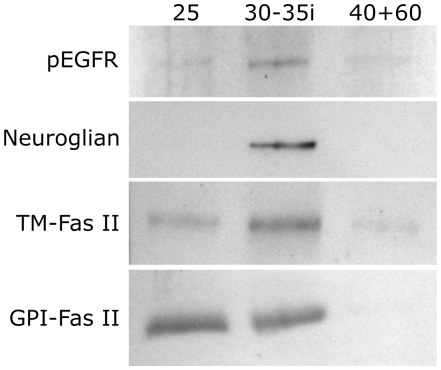
EGFR and IgCAM localization probed by sucrose gradient flotation of detergent resistant membranes. ALs were separated from brains and homogenized separately. Detergent-resistant and detergent-soluble membranes were separated by sucrose step-gradient flotation. Detergent resistant membranes were found in the 25% sucrose layer and at the 30−35% sucrose interface, while detergent soluble membranes were found in the 40 and 60% sucrose layers. Associated proteins were separated via PAGE and transferred to a PVDF membrane for immunoblotting. Using an antibody to activated EGFR, dimers (250 kDa) were found mostly in a detergent-resistant fraction at the 30−35% sucrose interface, smaller amounts were found in the 25% and 40+60% sucrose layers. For blots probed with an antibody to *M sexta* neuroglian, only the 30−35% sucrose interface fraction produced a band. As for the pEGFR blot, the TM-Fas II blot produced bands for all three fractions, with the 30−35% interface labeled more intensely than the other two fractions. An antibody to GPI-linked Fasciclin II, expected to be raft-associated by virtue of its GPI anchor, labeled only the 25% sucrose and the 30-35% sucrose interface fractions.

An alternative approach to identifying molecules associated with membrane rafts is to solubilize membranes with Triton X-100 at 4°C, then overlay with a sucrose gradient and centrifuge at high speed [76]. Triton-soluble membranes (phospholipid-rich) remain at or near the bottom of the gradient, while Triton-resistant membranes (rich in GSLs and sterols) float to a level corresponding to their lower density [76]. Fractions can then be collected and analyzed for components. We have previously done this with *M sexta* brains and found a Triton-resistant fraction containing GSLs, sphingomyelin, and a GPI-linked protein [63]. In the current study, *M sexta* brains were removed, antennal lobes separated from the rest of the brains, both tissues homogenized and subjected to Triton exposure, sucrose density flotation, PAGE separation and immunoblotting as previously described [63]. For the AL fractions, the pEGFR immunoblot revealed a strong band corresponding to EGFR dimers (250 kDa) at the 30−35% interface, with weaker bands at the 25% layer and the 40+60% (Triton-soluble) layers (Fig. 5), possibly an indication of EGFR movement in and out of rafts, as has been shown to occur with other RTKs [30], [35], [39]. The pattern for TM-Fas II resembled that for pEGFR, while bands for GPI-Fas II were seen only in the 25% fraction and the 30−35% interface, as expected for a GPI-anchored protein [76]. Neuroglian was seen only in the 30−35% interface, suggesting that it is found only in Triton-resistant membrane subdomains. Interestingly, similar blots performed on fractions isolated from the remainder of the brains from which the ALs were removed gave a different pattern, with pEGFR and TM-Fas II labeling primarily in the 25% and the 40+60% fractions, with stronger labeling in the latter (not shown). Labeling for neuroglian was primarily in the 25% sucrose fraction, with weaker labeling for the 30−35% interface and the 40+60% fraction (not shown). Thus it appears that the distribution of these molecules in various membrane subdomains differs with brain region, and that the TM proteins studied here are capable of moving between membrane subdomains. As for the ALs, brain GPI-Fas II was found in the 25% fraction and the 30−35% interface as expected.

### Sequestration of membrane sterols in vitro and in vivo

**Figure 6:**
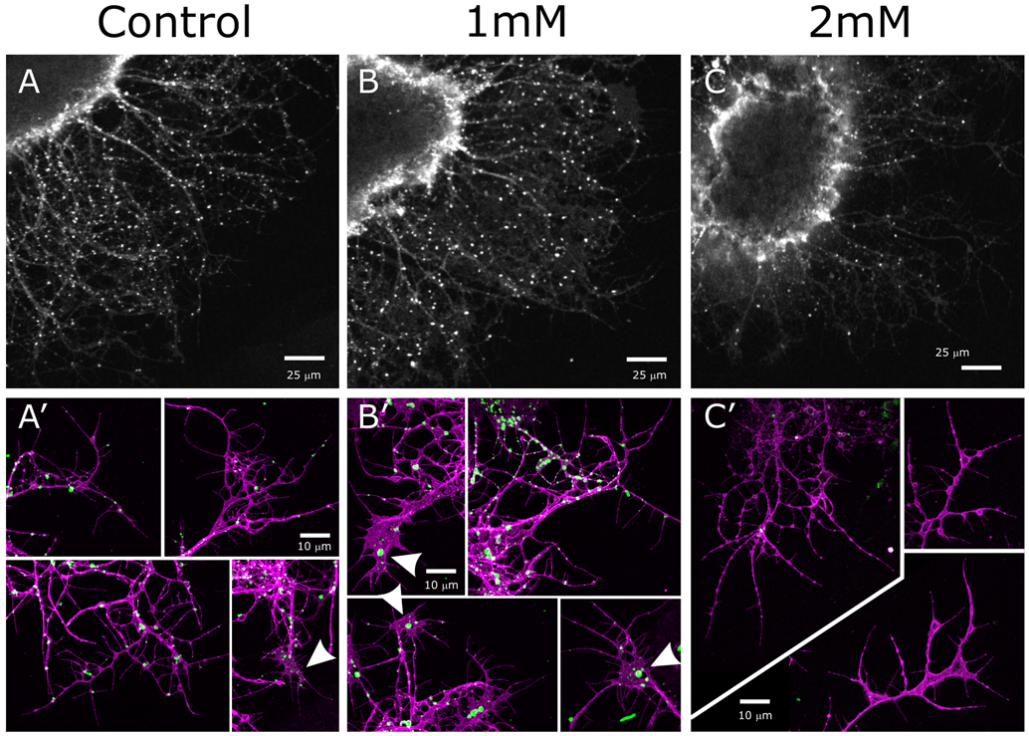
MβCD treatment alters the distribution of GSL-rich membrane subdomains. Control and MβCD-treated 30-hr ORN explant cultures were fixed and labeled with WGA (white in A–C; green in A′–C′) and an antibody to horseradish peroxidase (magenta in A′–C′) as a general neuronal marker. A, A′: As in previous figures, WGA labels small patches on axons in controls. Flattened growth cones exhibit very small patches (arrowhead in bottom right panel in A′). B: At 1 mM MβCD, there is a marked reduction in WGA labeling of axons. B′: Flattened growth cones exhibit larger WGA labeled patches (arrowheads), suggesting an aggregation of GSL-rich subdomains as sterols are removed. C, C′: At 2 mM MβCD, WGA labeling of axons is almost completely eliminated. C: WGA labeling persists in the cell bodies (bright labeling is edge of explant).

Methyl-β-cyclodextrin (MβCD) is a water-soluble, seven-membered ring of amylose molecules that forms complexes with sterols, which are a major component of all membrane rafts [19]. It has been used to extract sterols from the membranes of cells growing in culture as a way to study the cellular effect of disrupting membrane rafts [32], [33], [77]–[79]. ORN explants treated with MβCD displayed a dose-dependent decrease in the patchy WGA labeling of axon membranes (Fig. 6), suggesting that a drug known to disrupt membrane rafts by sequestering sterols also disrupts concentration of GSLs in patches. The persistence of WGA labeling in cell bodies (Fig. 6C) may indicate that GSLs are synthesized but not transported to the axons.

Studies using MβCD *in vitro* have provided useful insights, but until now no one has used MβCD to study effects *in vivo*. *M sexta* provides a tractable system in which to use MβCD to investigate the effects of disruption of raft-dependent signaling on development *in vivo*, where axons are navigating through their normal three-dimensional environment. Unlike most animals and plants, insects are unable to synthesize sterols *de novo* and must obtain them in their diet [80]. Because we injected animals during their pupal (and therefore non-feeding) phase, we expected that once sterols were extracted from membranes by MβCD they could not be replenished by *de novo* synthesis.

**Figure 7:**
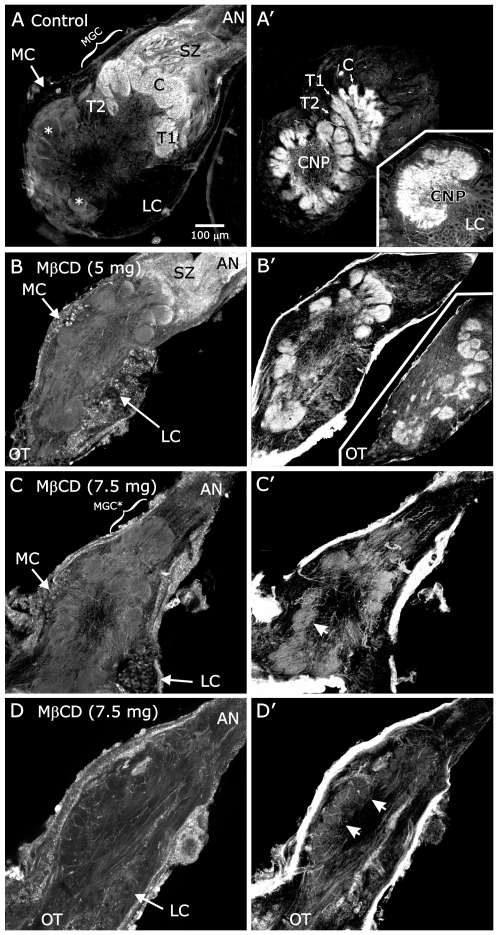
MβCD causes abnormal antennal lobe development. MβCD injection at early stage 3, animals allowed to develop to stage 14 (A–C) or 18 (D). Midline to the left. Brains were double labeled with WGA (A–D) and with Jacalin (A′–D′ plus insets). A: Control – ORN axons terminating in the male-specific macroglomerular complex (MGC, consisting of the Cumulus (C) and Toroids 1 & 2 (T1&T2)) label with WGA; axons terminating in the ordinary glomeruli (*) do not. A′: Jacalin-labeled AL neuron dendrites arborize in a glomerular pattern in both ordinary and MGC glomeruli. (CNP): coarse neuropil. A′ (inset): AL neuron dendrites in an untreated AL chronically deprived of ORN axon innervation have a diffuse, aglomerular arbor. B: 5 mg MβCD. Male-specific ORN axons retain some WGA labeling, but both MGC and ordinary glomerulus organization is perturbed and lobes elongate. Bright WGA labeling of the lateral and medial cell body clusters (LC, MC) is due to high WGA affinity for a nuclear membrane protein [40]. B′: Jacalin labeling highlights the disordered arrangement and lower number of glomeruli in treated animals. B′ (inset): In another animal injected with 5 mg MβCD, glomerulus-like structures appear even in the normally glomerulus-free coarse neuropil. C,D: 7.5 mg MβCD. Bright WGA labeling of MGC axons is completely lost though an MGC-like structure (MGC*) is present in panel C. C′,D′: Lobular structure of neuropil is faintly visible (arrowheads), but organization of the lobe is deeply perturbed despite the presence of substantial antennal nerves (AN). OT: output tracts. SZ: sorting zone region of the AN. Scale bar in A applies to all panels.

To test for the ability of MβCD to disrupt membrane rafts *in vivo*, we took advantage of the fact that GSLs are preferentially concentrated in these domains and that previous work had shown that WGA labels GSLs (rather than glycoproteins) on ORN axons in a developmentally regulated manner [40]. In that previous study, when no detergent was included in the labeling protocol, most, if not all, ORN axons of male and female brains were labeled with WGA along their lengths at stage 7, but in males, by adulthood, the label appeared only on the axons targeting the male-specific macroglomerular complex. In the current experiment, seven males at early stage 3 (prior to the arrival of ORN axons at the AL) were injected with 5 or 7.5 mg MβCD in insect saline and three were injected with the saline vehicle only. Animals were allowed to develop to stage 14 (when glomerular architecture has been well established and ORN axon ingrowth is complete; n = 3) or to stage18 (mature; n = 4). Brains were fixed, sectioned and labeled with WGA. Vehicle-injected control animals (stage-18 animals; n = 3) (Fig. 7A) exhibited the characteristic bright labeling of axons targeting the macroglomerular complex. In contrast, MβCD-treated animals (Fig. 7B–D) exhibited a dose-dependent reduction of WGA labeling in the macroglomerular complex, indicating that the GSL-rich membrane subdomains on ORN axons had been disrupted by treatment with MβCD *in vivo*, as was seen in ORNs treated with MβCD *in vitro* (Fig. 6). Treated animals exhibited no visually obvious indication of cytotoxicity, such as significant reduction in antennal nerve diameter [67; see below] or in the number of neuronal cell bodies associated with the AL.

MβCD treatment also disrupted the general structure of the AL. The ALs in treated animals often were elongated (Fig. 7B–D) and displaced laterally relative to their normal positions on the anterior surface of the protocerebrum flanking the midline. The glomerular organization of the ALs also was abnormal (Fig. 7B–D). This disruption was especially obvious when the AL neuropil was labeled with the lectin Jacalin, which binds to dendrites of AL neurons, most intensely in the basal regions of glomeruli [40]. Jacalin labeling of control male ALs (Fig. 7A′) reveals the typical pattern of organization in which the ordinary glomeruli form a layer surrounding a central coarse neuropil; the male-specific macroglomerular complex is located in the lateral region of the lobe near the entry site for the antennal nerve. In contrast, Jacalin labeling in MβCD-treated animals revealed a dose-dependent reduction in labeling intensity and a disorganized pattern of small, oddly shaped glomerulus-like structures (Fig. 7B′–D′); in some cases, they were located throughout the AL neuropil instead of in a defined glomerular layer (Fig. 7B′, inset). We could not identify particular ordinary glomeruli that normally are recognizable by their shapes and positions [81]–[83]. The macroglomerular complex also was smaller and more fragmented, lacking its typical lobular structure and the distinction among the three compartments that normally comprise it (Fig. 7B–D, B′–D′).

The pattern seen after MβCD treatment is clearly different from that seen when the developing AL is deprived of ORN axons [44], [66], [84]. In unafferented ALs, glomeruli are absent and the dendrites of AL neurons arborize in a diffuse layer around a coarse central neuropil (inset, Fig. 7A′). In the MβCD-treated animals, the antennal nerves were somewhat smaller in diameter than is normal (Fig. 7B–D). We considered whether this decrease, likely to reflect a diminution of the number of ORN axons reaching the AL, might be responsible for the altered neuropil structure in the MβCD-treated animals. In a previous study, we removed differing amounts of the antenna, to determine how many ORN axons are necessary for normal development of the glomerular array in the AL. We found that a slender nerve containing axons from only the proximal 21 antennal annuli (out of 70–80 total), comprising approximately 30% of the 300,000 ORNs normally innervating the AL, is sufficient to cause formation of the normal number of glomeruli, in a normal-appearing array [67]. Therefore, the diminution in diameter of the nerves seen in the MβCD-treated animals is not likely to be the cause of the abnormal neuropil architecture.

Thus, MβCD treatment resulted in loss of WGA-labeling, which we interpret to indicate the loss or dispersal of GSLs, and in abnormal AL architecture that could not be attributed simply to failure of axon ingrowth. An effect of MβCD mediated via projection neurons also seems unlikely, as previous experiments have shown that removal of 200 of the total 300 AL projection neurons, which leaves the dorsal glomeruli without projection neuron dendrites, does not affect the formation or stability of these glomeruli [85].

### Effect of MβCD treatment on EGFR and Ig-CAM immunolabeling

#### *M sexta* fasciclin II

*M sexta* fasciclin II (MFas II) is a homolog of vertebrate NCAM/RnCAM/OCAM [86], [87] and acts primarily as a homophilic adhesion molecule important in regulation of fasciculation as well as in synapse stabilization and plasticity [88]–[92] and neurite outgrowth [93]. In *M sexta*, Fas II exists in two forms, transmembrane and GPI-linked [55]. Studies have shown that TM-Fas II is expressed by a subset of ORN axons that targets a stereotyped array of 14–21 olfactory glomeruli [10]. The MFas II-expressing axons, which are diffusely scattered across the width of the antennal nerve as it leaves the antenna, assemble into large MFas II-positive bundles as they traverse the sorting zone. MβCD-treated (n = 27) and control (n = 12) female animals were allowed to develop to stage 6 so that we could analyze the behavior of ORN axons as they extended through the SZ and formed glomeruli. Because an *M sexta* AL is innervated by approximately 300,000 very small diameter ORN axons (∼0.1–0.3 µm) [94], use of general axon markers or lipophilic dyes would not have distinguished individual axons (as they can in *Drosophila*) and therefore would not have been useful (see EGFR and neuroglian labeling, below, for examples of general axon markers). As a practical alternative, a subset of axons was labeled using MFas II (C3) immunoreactivity. This approach allowed visualization of axonal outgrowth, trajectories, and fasciculation in the olfactory pathway, as well as assessment of possible effects on the expression of MFas II itself.

**Figure 8:**
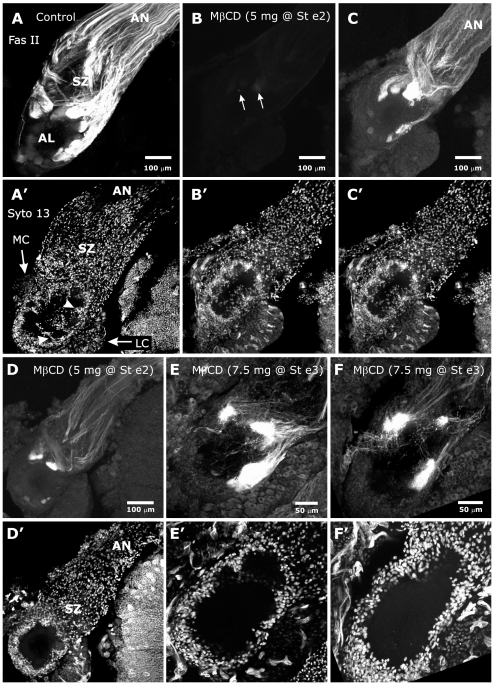
MβCD treatment dramatically reduces labeling for MFas II and perturbs the organization of MFas II-positive glomeruli. A: Control AL from a stage-6 animal labeled with anti-MFas II (C3). ORN axons undergo dramatic changes in fasciculation and trajectory as they traverse the sorting zone (SZ); MFas II-positive and -negative axons segregate into relatively large distinct fascicles as they exit the SZ. A′: The same section labeled with the nucleic acid dye Syto 13 to show cell nuclei. Neuropil glial-cell processes extend partially around developing glomeruli and some glial cell bodies migrate into the neuropil between glomeruli (arrowheads). MC and LC: medial and lateral clusters of AL neuron cell bodies. B: 5 mg MβCD. With collection parameters identical to those used in panel A, a stage-6 AL displays almost no visible MFas II labeling (brightest glomeruli are indicated by arrows). B′, C′: Neuropil glial migration is somewhat reduced. C–F: Increased gain settings for the MFas II channel to visualize axon behavior. C: MFas II-positive axons in same AL shown in panel B show changes in trajectory and fasciculation that typically occur in the SZ, but then form glomeruli more variably sized than in controls. The large Fas II-positive, tightly fasciculated bundles normally present as the axons exit the SZ are absent and the axons traveling in the nerve layer are less tightly bundled. D, D′: 5 mg MβCD. Glomeruli from another animal are also smaller in size. Neuropil glial cells show minimal migration, but SZ glial cells have migrated into the antennal nerve (AN). E,E′,F,F′: 7.5 mg MβCD. Methanol/formalin fixation, no Triton permeabilization. Gain settings for E and F were increased as in C and D to permit visualization of residual Fas II labeling. Glomeruli are small and irregularly shaped. Numerous axons extended laterally and centrally past the main body of the Fas II-positive glomerulus-like structures. Few NP glial cells migrated while SZ glial cells displayed robust migration.

In MβCD-treated animals, MFas II labeling was markedly reduced compared to controls (Fig. 8A, B). After viewing preparations at normal gain, we examined them again at higher gain to reveal weak MFas II labeling, so that we could assess the trajectories of MFas II-positive axons (Fig. 8C, D: 5 mg dose; Fig. 8E, F: 7.5 mg dose). These axons showed roughly typical behavior in the SZ, such as changing trajectory and forming MFas II-positive bundles as they exited the SZ, but many axons traveled either alone or in bundles far smaller and less discrete than usual (Fig. 8D–F). Within the AL, MFas II-positive axons normally target 14–21 glomeruli [10]. For the ALs of MβCD-treated animals shown in Figure 8, there were 13 and 18 MFas II-positive glomerulus-like axon terminations present at the 5-mg dose and 11 and 15 MFas II-positive terminations at the 7.5-mg dose, thus approaching or within the normal range. The MFas II-positive axon terminations in the treated ALs lacked the spheroidal shape of the typical developing glomerulus at this stage. Also, the borders of the glomerulus-like structures in treated ALs were less well defined; at higher magnification, many MFas II-positive axons were found to extend aberrantly from the sides of their glomeruli (Fig. 8F), a pattern normally seen only early in protoglomerulus formation [95]. Some axons extended from the bases of the glomerulus-like structures into the central coarse neuropil of the AL, which they also would not do normally (Fig. 8E, F).

Preparations were routinely double-labeled with the nucleic acid stain Syto 13 to visualize the pattern of glial nuclei in the SZ and around glomeruli (n = 64). SZ glia appeared to have migrated normally in MβCD-treated animals. In contrast, at both the 5 and 7.5-mg doses, very few neuropil glial cells had migrated to surround glomeruli (Fig. 8D′–F′).

Because of the irregular size and shape of glomeruli, the lack of a glial-cell surround, and the disrupted organization of the glomerular array in treated animals, we were unable to determine whether the glomeruli were in their correct positions within the ALs, and thus whether the MFas II-positive axons had targeted the correct positions. As in the animals examined at stages 14 or 18 (Fig. 7), antennal nerves were somewhat smaller in diameter, but we did not see overt signs of cytotoxicity, such as a reduction in the number of glial cells or AL neurons.

While MFas II labeling *in vivo* showed marked reduction in labeling intensity in the antennal nerve and in the AL, it was important to also examine the effect of MβCD treatment *in vitro*, which would reduce the impact of competing signals present in the normal 3D environment and which would allow clearer resolution of axons and growth cones. Our analysis of labeling was qualitative because the proportion of labeled axons in each explant varied and fasciculation of small groups of axons, a varying proportion of which were MFas II-positive, precluded quantification. Instead we systematically imaged each field in which there were labeled axons present at a density at which individual axons, small bundles of axons, and isolated growth cones could be resolved.

**Figure 9:**
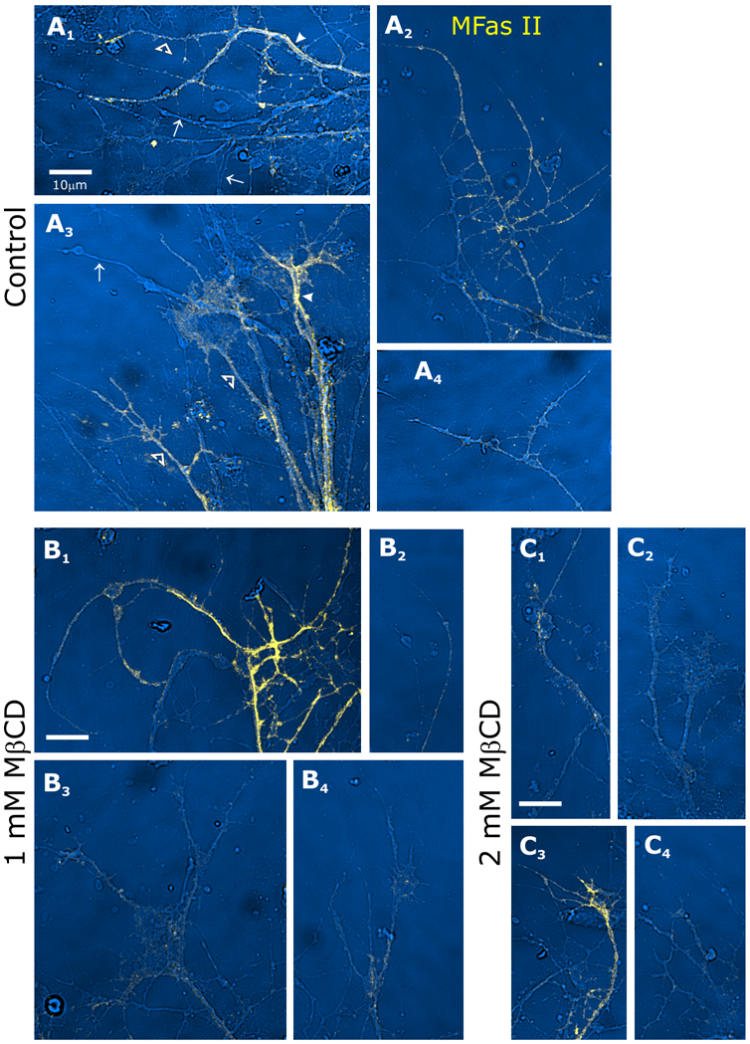
MβCD treatment of cultured ORNs decreases MFas II labeling. A_1–4_: In control conditions, a subset of ORN axons extending from explants are MFas II-positive, some strongly (arrowheads), some moderately (open arrowheads). Arrows indicate several unlabeled axons visible under brightfield optics. B_1–4_: After 24-hr exposure to 1mM MβCD, more MFas II axons are moderately or only weakly labeled. C_1–4_: At 2 mM MβCD, nearly all MFas II-positive axons are only faintly labeled. Rare axons that appear brightly labeled (C_3_) were always less strongly labeled than those found in control or 1 mM dishes. No consistent changes were seen in axonal or growth cone morphology at the 1 mM dose; axon outgrowth was reduced at the 2 mg/ml dose.

The set of images from control, and 1 and 2 mM MβCD dishes were compared. In control conditions, labeling for MFas II showed that a portion of the axons were MFas II-positive (Fig. 9A_1–4_), some strongly and some moderately, mirroring the *in vivo* staining pattern [10]. 24-hr exposure to MβCD visibly reduced labeling intensity. Strongly labeled MFas II axons were rare; most of the labeled axons were now only weakly labeled in the 2-mM dishes (Fig. 9C_1–4_). An intermediate pattern was visible in the 1-mM dishes (Fig. 9B_1–4_). We did not see major differences in the range of growth cone morphologies in the presence of MβCD, but did see reduced axonal length at the 2-mM dose.

#### Neuroglian

Neuroglian, an insect homolog of the vertebrate L1 [96], [97], is expressed in *M sexta* by both ORNs and glial cells [4; Oland, unpublished]. Neuroglian is present along the length of the ORN axons during most of the period of axon ingrowth, but is stabilized against extraction by detergent only in the SZ [4].

**Figure 10:**
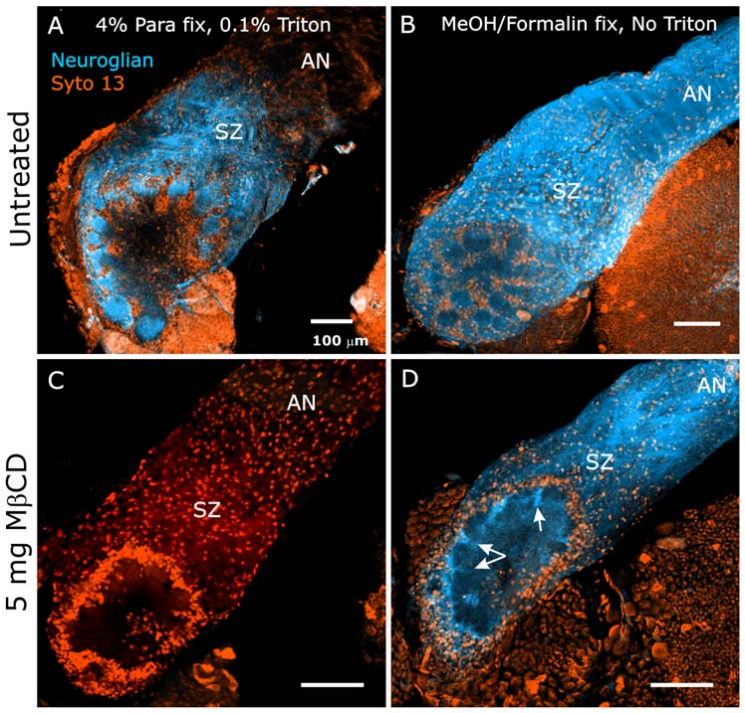
MβCD treatment decreases neuroglian stabilization. A,B: ALs of untreated brains show neuroglian labeling under two fixation paradigms. A: Standard paraformaldehyde fixation and Triton permeabilization produces labeling of ORN axons only in the SZ, the AL nerve layer and the glomerular layer, but not in the distal AN. B: Fixation with methanol/formalin demonstrates that neuroglian is also present distal to the SZ in the AN. C,D: 5 mg MβCD. Collection settings identical to those used in panels A and B, respectively. C: Standard fixation and permeabilization as in A. No neuroglian labeling is detected. D: Fixation with methanol/formalin. Neuroglian is present in the nerve and NP glial cells, though at lower-than- normal levels. D also clearly demonstrates that neuropil glial cells, while not migrating to surround glomeruli, did extend processes (arrows).

Because the *in vitro* co-localization experiments described earlier suggested that neuroglian could be found both in and outside of WGA-defined membrane domains, we labeled the brains of MβCD-treated and vehicle-injected control animals to determine the effect of MβCD on the disposition of neuroglian. We had found previously that the pattern of neuroglian labeling is dependent on the type of fixation/permeabilization used [4]. In the brains of control animals fixed with paraformaldehyde and permeabilized with Triton, an antibody against neuroglian labels ORN axons and glial cells in the SZ, axonal terminal branches in developing glomeruli, and glial processes surrounding glomeruli (Fig. 10A). In control brains fixed in methanol/formalin, which rapidly precipitates and fixes proteins in place, ORN axons also are labeled in the antennal nerve distal to the SZ (Fig. 10B), as they are in paraformaldehyde-fixed brains not permeabilized with Triton (not shown).

Note that the similarities in neuroglian labeling following two different fixation/permeabilization protocols (paraformaldehyde without Triton, and methanol/formalin) but not with paraformaldehyde + Triton, argues against an unmasking of epitopes distal to the SZ [4]. We have interpreted these differences in labeling with different protocols to mean that neuroglian molecules exist in two states along the axons of normal animals: a Triton-resistant state in the SZ and a Triton-extractable state distal to the SZ; the difference may be attributed to axon-glia interactions in the SZ that result in neuroglian stabilization via homophilic binding between cells and subsequent anchoring of the molecules to the cytoskeleton [4], as has been demonstrated for both L1 and neuroglian [98]–[102]. Because the relative contributions to resistance to Triton extraction made by homophilic binding and by anchoring to the cytoskeleton are not clear in *Manduca*, however, we use the word “stabilization” to encompass both of these possibilities.

When paraformaldehyde-fixed, Triton-permeabilized brains from MβCD-treated animals were examined, neuroglian was not detectable, indicating either that it was not present or that it had not been stabilized against Triton extraction during histological preparation, even in the SZ (Fig. 10C, n = 3). In methanol/formalin fixed tissue, however, neuroglian immunocytochemistry after MβCD treatment revealed labeling of ORN axons and of the glial cells that surround glomeruli (Fig. 10D, n = 3). Thus in MβCD-treated animals, neuroglian present on axons traversing the SZ does not become stabilized as it does in untreated animals, suggesting that neuroglian stabilization is linked to the existence of sterol-rich gMSDs.

As mentioned above [and see 4], neuropil glia also express neuroglian resistant to Triton extraction as they migrate to surround protoglomeruli. Brains of MβCD-treated animals processed using paraformaldehyde fixation/Triton permeabilization displayed no labeling for neuroglian (Fig. 10C), yet methanol/formalin fixation and neuroglian immunocytochemistry of MβCD-treated animals revealed that neuropil glia continued to express neuroglian and, though unable to migrate normally to surround protoglomeruli, were nevertheless able to extend their processes around developing glomeruli (Fig. 10D, arrows), suggesting that treatments designed to disrupt sterol-rich membrane subdomains disrupts signaling underlying cell migration but not process extension in one class of glial cells.

**Figure 11:**
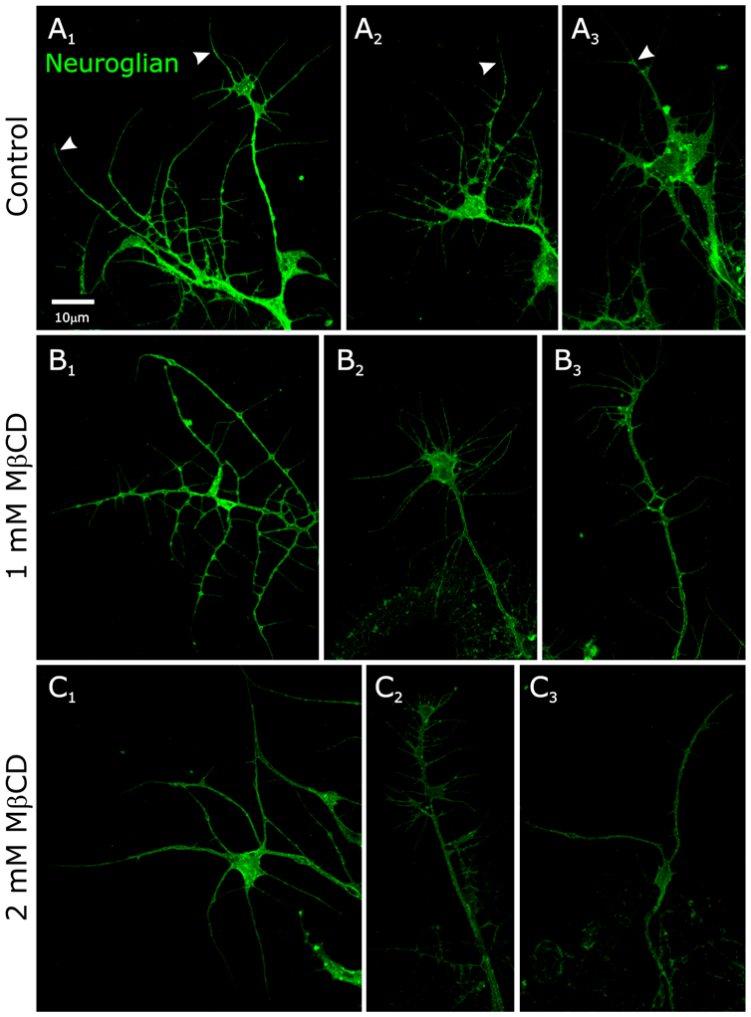
MβCD treatment modestly decreases neuroglian labeling in cultured ORN axons. Under control conditions (A_1–3_), neuroglian labeling appears along the length of the axons and in the growth cones, including the filopodia (arrowheads), and most axons were brightly labeled. After 24-hr exposure to 1 mM MβCD (B_1–3_), labeling intensity was somewhat reduced but the distribution of labeling along the axon and in growth cones was unchanged. Exposure to 2 mM MβCD (C_1–3_) resulted in a further reduction in labeling intensity across the population although some axons remained brightly labeled (C_1_).

Unlike the case for MFas II, neuroglian labeling is present on all axons in cultured ORNs, with the labeling generally quite uniform along the axon, as we find for ORN axons *in vivo* when not extracted with Triton. After treatment with 1 mM MβCD, there was a small but consistent decrease in labeling intensity (Fig. 11B_1–3_) in most axons, while those treated at the 2 mM level displayed a greater decrease (Fig. 11C_1–3_). A subset of the axons were strongly labeled in all cases, including the controls, but in the treated dishes, the incidence of strongly labeled axons decreased; at 2 mM, strongly labeled axons were rare (Fig. 11C_1_).

#### EGF Receptors

EGFRs are known to have a variety of developmental roles in vertebrates and invertebrates [103], [104]. In the moth olfactory pathway, EGFRs are expressed along the lengths of ORN axons throughout their extent, but are phosphorylated - a measure of activation [56] - in the sorting zone and in glomeruli as they become stable structures [4]. Since our *in vitro* results indicated that the EGFRs are localized to gMSDs, we tested whether disrupting the integrity of gMSDs with MβCD would disrupt EGFR signaling.

**Figure 12:**
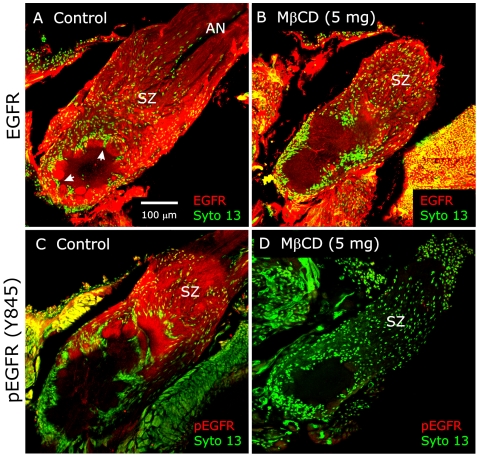
MβCD treatment blocks activation of EGF receptors. A,B: Labeling with an antibody to the EGF receptor (red) that recognizes the receptor regardless of activation state. The receptor is present on ORN axons in both control (A) and MβCD-treated (B) animals. Glial cells beginning to form envelopes (arrowheads) around protoglomeruli in panel A. C,D: Labeling with an antibody to the activated EGF receptor (red) shows labeling in the SZ and developing glomeruli in controls (C), but not in MβCD-treated animals (D).

Using an antibody to the EGFR that recognizes both the unphosphorylated and the phosphorylated forms, we found EGFRs to be present along the length of the ORN axons of MβCD-treated animals (n = 4), including in the SZ, just as they are in saline-injected controls (n = 2) (Fig. 12A, B). When we used an antibody that recognizes only the phosphorylated EGFR, however, we found the typical SZ and glomerular labeling of ORN axons in the saline controls (n = 6), but no detectable labeling in ORNs of MβCD-treated animals (n = 22) (Fig. 12C, D). Thus EGFRs are present, but do not appear to be activated in MβCD-treated animals.

MβCD would be expected to disrupt gMSDs on all cell types, and the function of any signaling molecule whose competence depends on association with these domains would have been compromised, not just the EGFRs and the IgCAMs addressed here. To help us understand whether MβCD blocks neuroglian stabilization by preventing EGFR activation, we took advantage of the facts that we had found previously that blocking EGFR activation with the specific inhibitor PD168393 [62] leads to loss of neuroglian labeling [4], and that several studies have linked homophilic binding of neuroglian/L1 molecules with EGFR activation and subsequent binding of neuroglian/L1 to the cytoskeleton [100], [105], [106]. Using the technique of alternate fixation/permeabilization shown in Figure 10, we asked if blocking EGFR activation alone, without disrupting membrane subdomains, could lead to loss of neuroglian stabilization, as opposed to loss of expression. Animals were treated with PD168393 at early stage 5 to block EGFR activation starting 2–3 days prior to stage 7, when Triton-resistant neuroglian labeling in the SZ is normally at a maximum. We compared DMSO-injected control animals (n = 4) and DMSO + PD168393-injected animals (n = 7), using both standard (4% paraformaldehyde with Triton permeabilization) and alternative (methanol/formalin, without Triton) fixation procedures for neuroglian.

**Figure 13:**
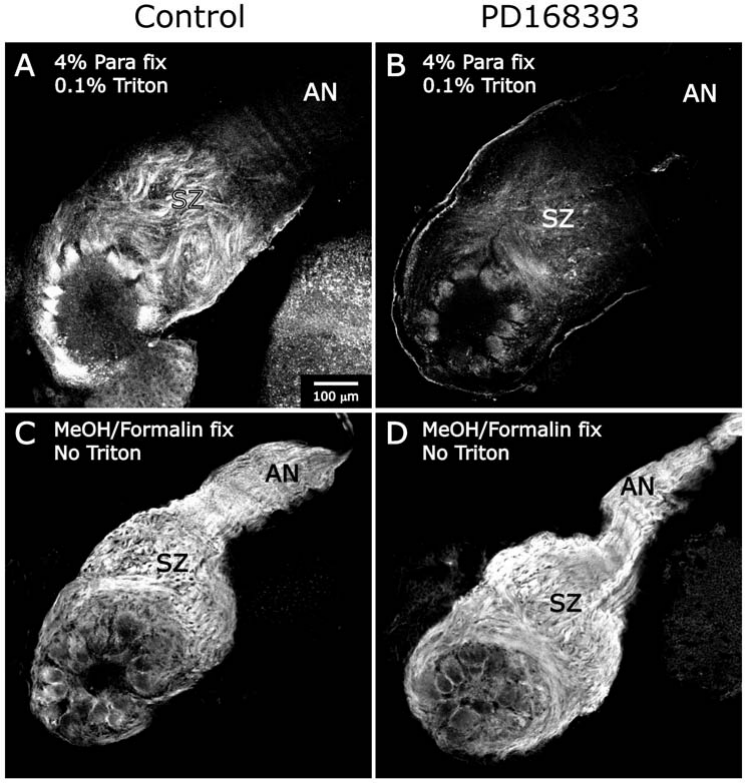
Blocking EGFR activation blocks neuroglian stabilization. Control (DMSO) and PD168393-treated animals injected at early stage 5. A: Control – paraformaldehyde fixation and Triton permeabilization. Neuroglian labeling typical of stage-7 olfactory pathway. B: PD168393-treated animals processed as in A. Much weaker labeling, typical of that normally seen at late stage 5 or early stage 6 [4]. C, D: Fixation/permeabilization by methanol/formalin results in strong neuroglian labeling in both controls (C) and treated animals (D), thus confirming the presence of neuroglian at normal levels. Gain settings were held constant in A and B, but decreased substantially for C and D because labeling was stronger under methanol/formalin fixation.

Standard immunocytochemistry of control brains (n = 2) produced the typical pattern of strong neuroglian labeling only in the SZ and in glomeruli (Fig. 13A), while treated brains (n = 3) displayed significantly reduced labeling, especially in the SZ (Fig. 13B). The neuroglian labeling that was visible in the treated animals examined at stage 7 was similar to that seen in untreated animals at stage 6 [4], suggesting that neuroglian that already had been stabilized at the time of blockade of EGFR activation remained so. In contrast, methanol/formalin fixation/permeabilization resulted in very similar levels of labeling for neuroglian in control (n = 2) and treated (n = 4) animals, (Fig. 13C, D), suggesting that neuroglian was present, but not stabilized, in ALs in which EGFR activation was blocked with PD168393. This evidence that prevention of EGFR activation also prevents neuroglian stabilization in the SZ suggests that MβCD's effect on neuroglian stabilization could, indeed, be an indirect effect via its effect on EGFR activation.

**Figure 14:**
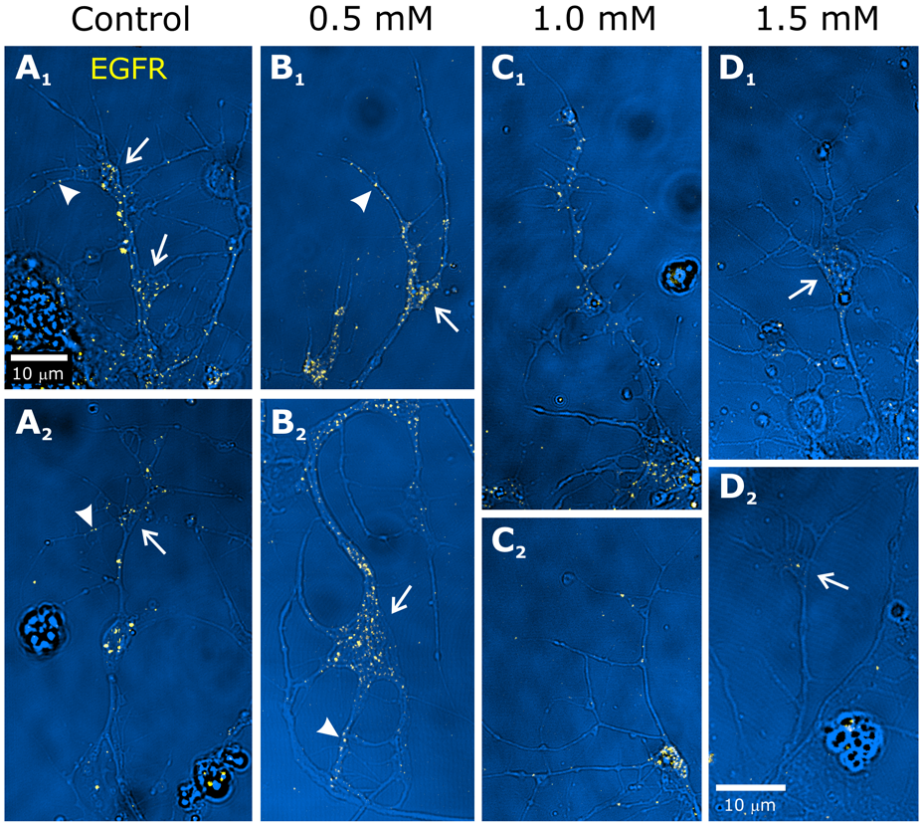
MβCD treatment of cultured ORNs decreases EGFR labeling. Under control conditions (A_1–2_), punctuate labeling for EGFRs (using antibody ab49966) appears along the length of the axons and in the growth cones (arrows), including the filopodia (arrowheads). ORNs exposed to 0.5 mM MβCD for 24 hours display no discernable changes in morphology or labeling for EGFRs (B_1–2_).After 24-hr exposure to 1 mM MβCD (C_1–2_), the punctuate labeling of filopodia and axons is reduced but labeling of flattened growth cones remains. Exposure to 1.5 mM MβCD (D_1–2_) results in nearly complete absence of labeling of axons and filopodia; weak labeling of some growth cones persists. Scale bar in A_1_ applies to all panels except D_2_.

As was the case for WGA, ORN explants treated with MβCD and labeled with an antibody to the EGFR exhibited a dose-dependant decrease in punctuate labeling of axons and growth cones (Fig. 14). At the 0.5 mM dose, axons, growth cones, and filopodia exhibited no change in labeling compared to controls (Fig. 14A_1–2_, B_1–2_). Explants treated with 1 mM MβCD displayed a decrease in axonal and filopodial labeling in some, but not all, ORNs (Fig. 14C_1–2_). Labeling of growth cones was not changed. At the 1.5 mM MβCD dose, however, all ORNs showed a significant decrease in labeling for the EGFR in axons, growth cones and filopodia (Fig. 14D_1–2_). The loss of punctuate EGFR labeling was not accompanied by a detectable increase in diffuse EGFR labeling. This result could be due either to loss of EGFRs or to an inability to detect EGFRs when they are not clustered. However, as shown above, EGFRs *were* detectable by immunocytochemistry after *in vivo* treatment, presumably because the signal from the hundreds of very fine ORN axons in a single optical section adds to reveal the EGFR label. It therefore appears that MβCD treatment results in dispersal of EGFR molecules in, rather than removal from, the membrane.

### Effect of MβCD treatment on axon targeting

**Figure 15:**
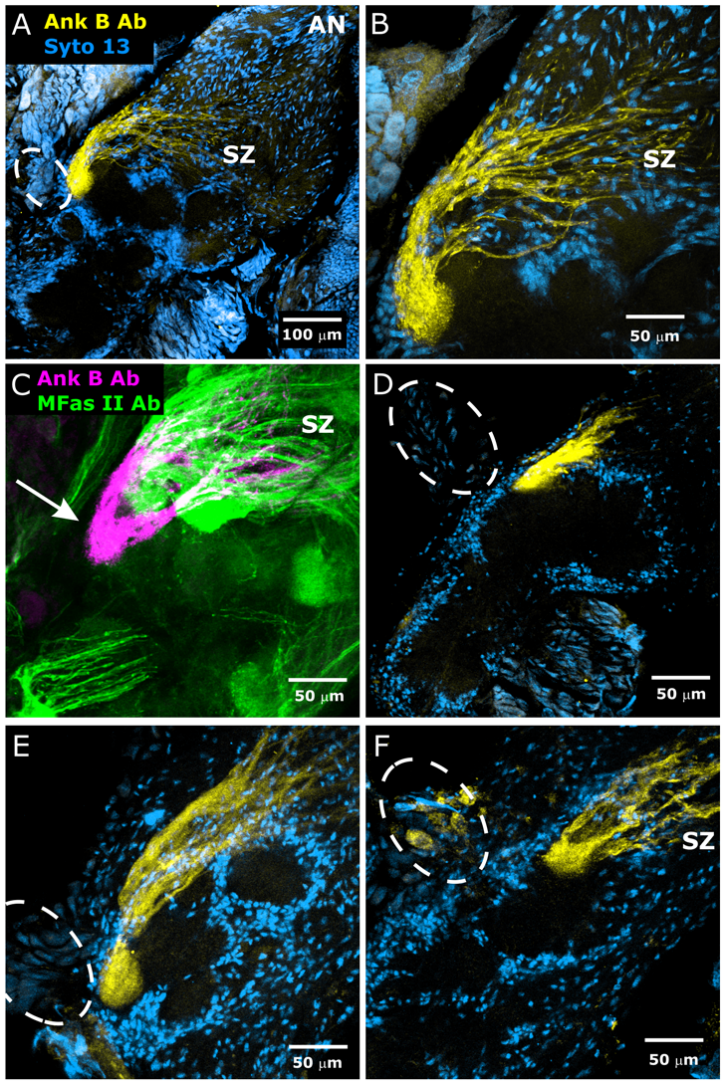
MβCD treatment does not prevent correct regional targeting of the axons innervating an identified glomerulus (glomerulus X). Untreated (A–C) and MβCD-treated (D–F) animals. A,B: Stage 7. An antibody to human ankyrin B (yellow) labels ORN axons targeting a single glomerulus located dorso-posteriorly close to primary neurites of the medial cluster of AL neurons (outlined with dashed line). Neuronal cell bodies and glial nuclei labeled with Syto 13 (blue). C: Double labeling with the ankyrin B antibody (magenta) and the MFas II antibody (green) demonstrates that glomerulus X (arrow) is Fas II-negative. D–F: 7.5 mg MβCD, injected at early stage 3, brain dissected at stage 7. Ank B axons target a single glomerulus located near the primary neurites of the medial cluster of AL neurons although the shape of the glomerulus is variable and the pattern of fasciculation in the SZ is somewhat abnormal.

Sequestration of membrane sterols clearly leads to abnormalities in ORN axon outgrowth, glomerulus formation, and migration of neuropil glia, and the abnormal patterns of MFas II-positive ORN axons visualized in MβCD-treated preparations hinted at the possibility of targeting defects (Fig. 8). To ask directly whether MβCD-treatment leads to mistargeting of ORN axons, we examined a set of ORN axons that normally target a single glomerulus (Fig. 15A,B). These axons label with an antibody to human Ankyrin B, though western blots of *M sexta* brains indicate that the epitope recognized is not a homolog of Ankyrin B (data not shown). For the purposes here, the precise nature of the epitope is not important; the antibody is useful here because of the specificity of labeling for ORN axons that target a unique glomerulus (we will call it “glomerulus X”), from animal to animal.

Glomerulus X is located dorsally and posteriorly, adjacent to the tract of primary neurites emanating from the medial cluster of AL neuronal cell bodies. The axons that contribute to the glomerulus are gathered in the SZ from across the width of the antennal nerve to form one large fascicle and a few smaller ones that target the glomerulus (Fig. 15A, B). Double-labeling experiments in normal ALs using the Ankyrin B antibody and the MFas II antibody revealed that glomerulus X is consistently one of the MFas II-negative glomeruli (Fig. 15C).

In animals treated with MβCD and processed for Ankyrin B immunocytochemistry (n = 11), we found glomerulus X to be present in roughly the correct position (adjacent to the primary neurite tract of the medial cluster), although it was impossible to say whether its position was completely normal due to the MβCD-induced disorganization and elongation of the antennal lobe (Fig. 15D–F). There was no indication that the axons extended beyond the boundaries of the target glomerulus either laterally or medially as was seen in the MFas II-positive glomeruli in treated animals (Fig. 8E, F); all of the labeled axons terminated in a well-defined glomerulus.

## Discussion

The results presented here show that subdomains in the membranes of ORN axons in the moth *M sexta* include the receptor tyrosine kinase EGFR and the IgCAMs neuroglian and fasciclin II. MβCD treatments designed to disrupt sterol-rich gMSDs *in vivo* result in loss of EGFR activation and of neuroglian stabilization in the SZ region of the olfactory pathway, and in significant decrease in fasciclin II expression by ORN axons. MβCD treatment during the period of ORN axon ingrowth affects not only the spatial organization of the glomerular array in the AL, but also the behavior of ORN axons as they grow through the SZ and form terminal arborizations in the AL. Migration, but not process extension, by neuropil glia is blocked following MβCD treatment, while proliferation and migration of SZ glia appears unaffected.

### Distribution of EGFRs, IgCAMs and GSL-rich subdomains in the membranes of ORN axons

In previous work [40], we showed that each of the major cellular elements of the primary olfactory pathway - ORNs, glial cells, and AL neurons - has a distinctive lectin-binding signature in the developing and adult animal. In particular, the ORN axons express one or more glycosphingolipids (GSLs) that are recognized by WGA. Sucrose-gradient flotation revealed a detergent-resistant membrane fraction that contained sphingomyelin, an important component of membrane rafts, as well as a GPI-anchored isoform of MFas II that would be expected to associate with membrane rafts [63]. In the current study, we have shown that labels for molecules expected or known to partition into membrane rafts are often distributed within fixed, cultured ORN axon membranes in a punctate pattern that is consistent with a distribution within rafts or platforms.

Our *in vitro* colocalization studies suggested that EGFRs are almost always associated with domains that label with WGA, while sucrose gradient flotation of detergent-resistant membranes, followed by western blots using an antibody to phosphorylated EGFRs suggested that EGFRs exist both in and out of these domains. The apparent discrepancy may be due to the very function hypothesized for rafts and platforms, that of concentrating certain molecules in closer proximity than they might be in the typical lipid bilayer [21], [24], [25], [29]. Thus the lack of detectable labeling outside of the GSL-rich domains could simply be due to greater distance between EGFR molecules, resulting in a diffuse, difficult to detect, labeling pattern. Alternatively, EGFR molecules in GSL-rich domains might move into phospholipid domains during the Triton solubilization and sucrose flotation process due to the lack of a GPI anchor [107]. Similar *in vitro* colocalization studies suggested that neuroglian and TM-MFas II are found in and outside of the WGA-labeled gMSDs, while immunoblots suggest that they are localized exclusively (neuroglian) or primarily (TM-Fas II) to Triton-resistant membrane subdomains. In light of our finding that WGA labels only a subset of Triton-resistant subdomains visualized with Vybrant DiI, these results suggest that neuroglian and TM-Fas II are found in multiple gMSDs of varying composition, only some of which label with WGA. In addition, it is possible that the localization of these IgCAMs depends on their homophilic binding in *trans* or multimerization in *cis* [37], [87], [105]. Transmembrane proteins are also known to move in and out of membrane subdomains based on fluctuations in membrane elasticity [26], [108], [109] or movement of other molecules into or out of those domains (see e.g., [110]).

### Disruption of GSL-rich membrane subdomains and effects on organization of the olfactory pathway

#### Is there a non-specific effect on ORN axon growth?

The diminution of WGA labeling in male-specific adult axons terminating in the MGC indicates that the GSL ligands were present in decreasing amounts after treatment with MβCD and suggests that gMSDs had been disrupted by sequestration of sterols. A possible consequence might have been a general effect on process outgrowth via disruption of interactions between gMSD-associated signaling molecules and downstream effectors that mediate changes in cytoskeletal organization or attachment to cytoskeletal elements. There is some evidence that MβCD also affects non-raft membrane domains by extracting sterols there as well; a few studies have reported that MβCD extracts a significant amount of phospholipids, though others find little or no extraction [111]. *In vivo*, we used doses (5 and 7.5 mg) estimated to produce circulating MβCD concentrations of 1.9 and 2.8 mM (extrapolated from Morton and Truman [112] using an estimated hemolymph volume of 2 ml), placing them at the low end of the range of doses used in previously reported cell culture experiments (0.5–20 mM; [111]). At the doses used here, most ORN axons continued to extend into the AL, the dendrites of AL neurons sent processes into glomeruli, SZ glia migrated normally, and neuropil glia extended processes into the neuropil. Our *in vitro* doses, 0.5 to 2 mM, reduced outgrowth only at the 2-mM level. Together these results suggest that the effects of treatment on the various elements of the olfactory pathway were not simply non-specific effects on growth due to generalized membrane disruption.

#### How does MβCD treatment affect neuron-glia interactions in the developing olfactory pathway?

Because we are interested in understanding the role of raft-based signaling as it affects interactions between ORN axons and between ORN axons and glial cells, we examined the ORN axons and glial cells in each region of the olfactory pathway to determine in what way disruption of membrane subdomains had affected them.

Normal ORN axons behave differently in each region of the developing olfactory pathway, and each region has a specific population of glial cells [9], [44], [113]. In previous studies, we documented at the cellular level several important interactions between neurons and glial cells, including: (1) populating the SZ by ORN-axon induction of proliferation and migration of central glial cells that lie at the entry to the AL; (2) glia-dependent regulation of axonal fasciculation in the SZ; (3) ORN axon-induced activation of neuropil glial-cell migration and process extension around developing glomeruli; and (4) stabilization of axonal protoglomeruli by neuropil glial cells [3].

MβCD treatment initiated at stage 2 had little, if any, effect on glial-cell migration into the SZ. MFas II-positive ORN axons within the SZ, however, displayed significantly reduced fasciculation, especially at the 7.5 mg dose (Fig. 8E, F). The fascicles of MFas II-positive axons were less robust at both dosages, but because the MFas II labeling was so diminished, we were unable to determine if MFas II-positive axons were bundled only with their normal MFas II-positive partners as they left the SZ.

The organization of the AL neuropil in MβCD-treated animals was disrupted in several ways that suggest that gMSD-based signaling affects several of the cellular interactions involved in development of the antennal lobe. First, the organization of the glomerular array was abnormal. In males, the typical morphology of the macroglomerular complex was lost and its size decreased. In animals in which ordinary glomeruli formed, they were oddly shaped, and distributed abnormally within the neuropil.

Second, the cell bodies of neuropil glia failed to migrate toward the center of the neuropil to surround and stabilize protoglomeruli [67], though they did extend processes. Glial cell process extension and migration normally is induced by the arrival of ORN axons [44]. The possibility exists that their failure to migrate in treated animals is the result of disruption of upstream processes critical to normal development, but the extension of neuropil glial processes, a requisite precursor to migration, suggests that initial signaling between glia and ORN axon terminals occurred normally. Thus it seems likely that the effects of MβCD treatment were on internal mechanisms by which migration of the glial cell body is coupled to process extension [e.g., 114].

In earlier studies we saw that glial processes alone can maintain glomerular structure into adulthood [1]. On the other hand, the failure to restrict the terminal branches of some axons (e.g., MFas II-positive axons, Fig. 8E–F) to their glomerular territory may reflect either less robust glial envelopes around each glomerulus or loss of gMSD-based signaling between glial processes and ORN growth cones that restricts axon terminal branches from crossing the glial borders between glomeruli. We found that the MFas II-positive axons displayed aberrant projections extending across glomerular borders while the axons targeting the MFas II-negative “glomerulus X” in MβCD-treated animals did not. This finding suggests that subsets of axons differentially weight the signals presented by the glia forming glomerular borders and by the axons with whom they traveled to their target.

Third, the dendrites of normal AL neurons have characteristically glomerular arbors. In the MβCD-treated animals, however, AL neurons exhibited a dendritic phenotype intermediate between the normal glomerular morphology and the diffuse, aglomerular arbors typical of AL neurons developing in ALs in the absence of ORNs ([44], [65], [84]; reflected in Fig. 7). As was the case for the ORN axons, the abnormal morphology of the AL dendrites could be a consequence of the loss of a robust glial envelope around developing glomeruli, itself possibly a consequence of loss of gMSD-based signaling interactions directly between ORN axons and AL neuron dendrites. It cannot be due to an insufficient number of ORN axons to induce glomerulus formation, however, because we previously showed that over 30% of ORN axons must be removed before glomerulus number is decreased, while greater reductions, up to 88%, will decrease the number of glomeruli but not the distribution of the glomeruli within the neuropil. Only at more than 88% reduction do we see loss of glomerular organization [67].

Taken as a whole, our results suggest that gMSD-based signaling may be critical to normal development of the AL via effects on neuron-neuron and/or neuron-glia signaling, some of which are likely to be the consequence of loss of EGFR-based signaling.

Importantly, our results also suggest that several of the cellular changes that occur as the olfactory pathway develops do *not* depend on gMSD-based interactions, regardless of the underlying molecular players. These include extension of neuropil glial-cell processes around developing glomeruli and migration of SZ glia into the entry region of the antennal nerve to populate the SZ. Our limited assessment of the accuracy of ORN targeting, using labeling of male-specific axons and the axons targeting glomerulus X, indicated that ORNs targeted approximately the correct region of the AL. Their ability to do so suggests that at least some ORN axons do not depend on gMSD-based signals to navigate to the correct region of the lobe.

### Disruption of membrane subdomains and effects on EGFR and the IgCAMS neuroglian and fasciclin II

#### To what extent did membrane subdomain disruption specifically affect expression and function of the IgCAMs and the RTKs?

To better understand the relationship between gMSDs and both the IgCAMs and the EGFR, we wanted to determine whether a molecular interaction between the CAMs and the EGFR initiates signaling cascades that underlie the axonal and glial interactions that drive ORN axonal navigation and sorting, and glomerulus formation and stabilization. Vertebrate EGFRs are activated by a wide range of ligands (EGF, TGF-α, heparin-binding EGF, betacellulin, amphiregulin, epiregulin, epigen and neuregulins; [115]). Insects appear to lack an EGF and rely instead on a number of TGF-α and neuregulin homologs (spitz, vein, etc.) for ligand-mediated activation of the EGFRs [103]. Growing evidence in insects suggests that RTK activation also can be mediated by homophilic IgCAM interactions in *cis* and in *trans* [93], [105], [116], [117]. Activation of these receptors in turn affects stabilization of the IgCAMs within the membrane [4], [101], [106]. Previous experiments in other systems in which EGFR activation, or fasciclin II or neuroglian expression was specifically blocked pharmacologically or by genetic manipulation have revealed axon stalling and abnormal fasciculation phenotypes [86], [116], [118], [119]. Similarly, blocking EGFR activation in *M sexta* leads to fasciculation defects and axon stalling in the SZ, as well as to loss of neuroglian stabilization ([4; present study], thus also supporting a functional link between EGFR activation and neuroglian binding and stabilization. If, as the current study suggests, the molecular interaction occurs in association with a membrane subdomain, we can expect to find that activation of the EGFR induces activity in molecular partners also associated with such a subdomain.

The current study contributes three important pieces of information supporting a link between EGFR function, neuroglian stabilization, and an involvement of membrane subdomains in EGFR and neuroglian function and fasciclin II expression or transport.

First, activation of EGFRs on ORN axons of *M sexta*, which normally occurs only in the SZ and in developing glomeruli [4], appears to require intact, sterol-rich membrane subdomains. Our *in vitro* results indicate that the presence of visible clusters of EGFRs in ORN axons is reduced after disruption of membrane subdomains while our *in vivo* results indicate that EGFRs are present, but not activated, following disruption of subdomains with MβCD.

It is important to stress, however, that our results should not be interpreted to suggest a widescale blockade of EGFR activation, as would be expected for PD168393 treatment. We would expect that the EGFR activation throughout the brain that is important for cell differentiation, migration, and survival [103] would still be accomplished via the traditional ligand-mediated avenues (TGFα and neuregulin families), and that it is the *ligand-independent* activation pathway, caused by multimerization of IgCAMs binding in *trans* and in *cis* [105], that was affected. This would explain the absence of detectable cell death and the phenotypes of perturbed axon fasciculation and loss of NP glial migration, processes which would require functional coupling of membrane-bound IgCAMs to the cytoskeleton.

Second, labeling for detergent-resistant neuroglian, which normally is found in ORN axons only from their entry into the SZ until they penetrate the neuropil glial cell layer to form protoglomeruli [4], was eliminated in MβCD-treated animals although neuroglian remained present along ORN axons, as shown following fixation with methanol-formalin. *In vitro* treatment with MβCD, which allowed examination of labeling in individual axons, showed there to be a visually obvious but relatively small decrease in labeling intensity across the population of ORN axons. Based on the combination of *in vivo* and *in vitro* results, the latter similar to those seen for L1 in cultured hippocampal neurons treated with the raft-disrupting agent fumonisin B1 [120], we conclude that disruption of membrane subdomains reduces but does not prevent trafficking of neuroglian along the ORN axons, but does eliminate stabilization of neuroglian in the SZ [4].

Third, in *M sexta* treated with PD168393, which irreversibly inactivates the kinase function of the EGFR, but not other protein kinases ([62]), Triton-resistant neuroglian labeling of ORN axons is virtually eliminated, though alternate fixation/permeabilization processing shows neuroglian to be present at normal levels [4; current study]. These results suggest that EGFR activation is essential for neuroglian stabilization and are consistent with the results of Sepp and Auld [119], who reported that expression of a dominant-negative EGFR (kinase dead) in *Drosophila* decreased “expression” of neuroglian by glial cells (as determined by immunocytochemistry using Tween 20).

Our current results indicate that neuroglian exists both in and out of WGA-labelled gMSDs (explant cultures), but exists only in Triton-resistant fractions (sucrose gradient-western blots), and thus must also exist in gMSDs that do not label with WGA (see also [40]). Together these results suggest that interactions between neuroglian and the EGFR could depend in part on regulating neuroglian partitioning between the EGFR-containing gMSDs that label with WGA and gMSDs that do not label with WGA and do not contain EGFRs.

Additional experiments will be needed to determine the extent to which the absence of neuroglian stabilization following MβCD treatment is due to a) perturbation of homophilic neuroglian binding in *cis* and *trans* (previously shown to activate EGFRs; [105], [116]), b) loss of activation of a component of the EGFR to MAP kinase signaling pathway (shown to lead to attachment of L1 to the cytoskeleton; [100], [106]) or c) perturbation of neuroglian's ability to link to the cytoskeleton by recruiting or binding to molecules such as ankyrin, doublecortin, or members of the ezrin-radixin-moesin family [100], [105], [106], [121]–[132]. Nevertheless, the current experiments suggest that gMSDs are essential for ligand-independent EGFR activation and for anchoring of neuroglian to the cytoskeleton, both required for normal extension and sorting of ORN axons.

In contrast to our results for neuroglian, in which MβCD treatment altered the stabilization of the moleculesand produced only a small decrease in the apparent amount of the molecules but not in their distribution, our immunocytochemical results with MFas II both *in vivo* and *in vitro* suggest that its presence on ORN axons was significantly reduced. Our culture experiments suggest that MFas II is rarely associated with WGA-labeled membrane domains, but the Vybrant DiI-WGA co-labeling experiments suggest the presence of another class of gMSDs with which the MFas II could be associated. The sucrose gradient flotation experiments, in which TM-Fas II was found primarily in Triton-resistant fractions, support this scenario. Rafts are known to be one of a variety of mechanisms used in the transport of certain molecules along axons [22], [133]–[135], but some of these molecules, neuroglian for example, appear to have access to multiple mechanisms of axon transport [136] and thus would continue to be transported after MbCD treatment. Perhaps TM-MFas II depends solely on raft-like membrane domains for transport and thus would become depleted in the face of MbCD treatment.

### Summary

In previous studies, we have shown that both axon-axon and axon-glia interactions operate at several points in the developing olfactory pathway to ensure that ORN axons sort, form fascicles, and navigate toward their correct glomerular target, and that glomerulus construction and stabilization proceed properly. The results of studies of IgCAM-RTK-cytoskeleton interactions in both vertebrate and invertebrate systems suggest that binding between L1/neuroglian molecules in *trans* and in *cis* results in EGFR activation via a ligand-independent mechanism which in turn stabilizes the neuroglian, presumably by activating a pathway that links it to the cytoskeleton. The current results in the *M sexta* olfactory pathway indicate that many EGFRs are distributed within membrane subdomains and that both EGFR activation and neuroglian stabilization are reduced when these subdomains are disrupted. Removal of membrane sterols by treatment with MβCD affects ORN axon growth and fasciculation in the sorting zone region of the antennal nerve, affects the organization and distribution of glomeruli, and allows axon terminal branches to extend beyond their glomerular territory, at least in a subset of ORN axons. Together, the results are consistent with a functional link between EGFR activation and neuroglian stabilization, possibly mediated by their clustering in GSL-rich membrane subdomains. In addition, the results require that any model of the primary and downstream interactions occurring in this system take into account both the dynamic nature of gMSD-based interactions and the molecular players likely to be associated with these subdomains. It is not yet clear whether the role of the subdomains in modulating the neuroglian-EGFR interaction is to facilitate translation of EGFR activity into activation of downstream pathways or whether they play a direct role in mediating neuroglian-neuroglian and/or neuroglian-EGFR interactions.
